# Chemical Constituents with GNMT-Promoter-Enhancing and NRF2-Reduction Activities from Taiwan Agarwood *Excoecaria formosana*

**DOI:** 10.3390/molecules25071746

**Published:** 2020-04-10

**Authors:** Ho-Cheng Wu, Ming-Jen Cheng, Chia-Hung Yen, Yi-Ming Arthur Chen, Yi-Siao Chen, Ih-Sheng Chen, Hsun-Shuo Chang

**Affiliations:** 1 Graduate Institute of Natural Products, College of Pharmacy, Kaohsiung Medical University, Kaohsiung 807, Taiwan; duncanwu762001@gmail.com (H.-C.W.); chyen@kmu.edu.tw (C.-H.Y.); 2 Bioresource Collection and Research Center (BCRC), Food Industry Research and Development Institute (FIRDI), Hsinchu 300, Taiwan; cmj@firdi.org.tw; 3 Drug Development and Value Creation Research Center, Kaohsiung Medical University, Kaohsiung 807, Taiwan; 4 Master Program in Clinical Pharmacogenomics and Pharmacoproteomics, College of Pharmacy, Taipei Medical University, Taipei 110, Taiwan; arthur@tmu.edu.tw; 5 Department of Medical Research and Education, Cheng Hsin General Hospital, Taipei 112, Taiwan; 6 Ph.D. Program in Environmental and Occupational Medicine, College of Medicine, Kaohsiung Medical University, Kaohsiung 807, Taiwan; dragonraja7992@yahoo.com.tw; 7 School of Pharmacy, College of Pharmacy, Kaohsiung Medical University, Kaohsiung 807, Taiwan; m635013@kmu.edu.tw; 8 Department of Medical Research, Kaohsiung Medical University Hospital, Kaohsiung 807, Taiwan

**Keywords:** *Excoecaria formosana*, Euphorbiaceae, secondary metabolites, GNMT promoter activity, NRF2 activity

## Abstract

Hepatocellular carcinoma (HCC) is considered to be a silent killer, and was the fourth leading global cause of cancer deaths in 2018. For now, sorafenib is the only approved drug for advanced HCC treatment. The introduction of additional chemopreventive agents and/or adjuvant therapies may be helpful for the treatment of HCC. After screening 3000 methanolic extracts from the Formosan plant extract bank, *Excoecaria formosana* showed glycine *N*-methyltransferase (GNMT)-promoter-enhancing and nuclear factor erythroid 2-related factor 2 (NRF2)-suppressing activities. Further, the investigation of the whole plant of *E. formosana* led to the isolation of a new steroid, 7α-hydroperoxysitosterol-3-*O*-β-d-(6-*O*-palmitoyl)glucopyranoside (**1**); two new coumarinolignans, excoecoumarin A (**2**) and excoecoumarin B (**3**); a new diterpene, excoeterpenol A (**4**); and 40 known compounds (**5**–**44**). Among them, Compounds **38** and **40**–**44** at a 100 μM concentration showed a 2.97 ± 0.27-, 3.17 ± 1.03-, 2.73 ± 0.23-, 2.63 ± 0.14-, 6.57 ± 0.13-, and 2.62 ± 0.05-fold increase in GNMT promoter activity, respectively. In addition, Compounds **40** and **43** could reduce NRF2 activity, a transcription factor associated with drug resistance, in Huh7 cells with relative activity of 33.1 ± 0.2% and 45.2 ± 2.5%. These results provided the basis for the utilization of Taiwan agarwood for the development of anti-HCC agents.

## 1. Introduction

Hepatocellular carcinoma (HCC) is the sixth most commonly diagnosed cancer, and was the fourth global leading cause of cancer mortality in 2018 [1]. It is typically an aggressive tumor that arises in the setting of underlying chronic liver disease in most cases. Liver cirrhosis caused by long-term damage remains the most important risk factor for the development of HCC, regardless of etiology. Other risk factors, such as alcohol abuse, hepatitis B and C, diabetes, and nonalcoholic fatty liver disease, also contribute to HCC [2]. Until now, there are some available treatment options for HCC patients, including surgical resection, liver transplantation, chemotherapy, and targeted cancer therapy [2]. Unfortunately, all of these HCC management types have several drawbacks and limitations [2,3]. Although the preferred therapy is surgical resection, the tumor size, health status, and cancer stage of HCC patients need to be carefully considered. Liver transplantation is the standard of care for HCC patients, but available liver donors and transplant rejection are major limitations of this therapy option. Chemotherapy for HCC is known to induce drug resistance and have a range of adverse effects. The cost, toxicity, drug resistance, and drug-design difficulty are disadvantages of targeted cancer therapy such as sorafenib. For now, the introduction of additional chemopreventive agents and/or adjuvant therapies is a new strategy that may be helpful for the treatment of HCC. Chemoprevention refers to the use of substances such as hormones, medications, diet-related agents, and vaccines to stop cancer from developing [4]. Overexpression of glycine *N*-methyltransferase (GNMT) has been shown to decrease DNA adduct formation and cytotoxicity induced by carcinogens [5,6,7]. Previously, a GNMT inducer was identified by a high-throughput screen, and has been proven to have an antitumorigenic effect against HCC cells. Thus GNMT inducers have been considered to be chemopreventive agents [8]. Adjuvant therapy is given additional cancer treatment to the primary or initial therapy, which can lower the risk of cancer recurrence or help to reach the ultimate goal [9]. Nuclear factor erythroid 2-related factor 2 (NRF2) is a basic leucine zipper (bZIP) transcription factor. As evidenced by many studies, increased expression of NRF2 is associated with HCC development [10]. Thus, reducing NRF2 expression activity in cancer cells can suppress HCC cell growth, reverse the chemotherapeutic resistance of HCC, and enhance the sensitivity of cancer cells to anticancer drugs [10]. Having the intention of identifying anti-HCC agents, we screened two platforms with 3000 methanolic extracts from the library of Formosan plants in the Laboratory of Medicinal Botany of Kaohsiung Medical University [11]. The prepared extracts from *Excoecaria formosana* Hayata (Euphorbiaceae family) were identified to have GNMT-promoter-enhancing and NRF2-suppressing activities.

**Figure 1:**
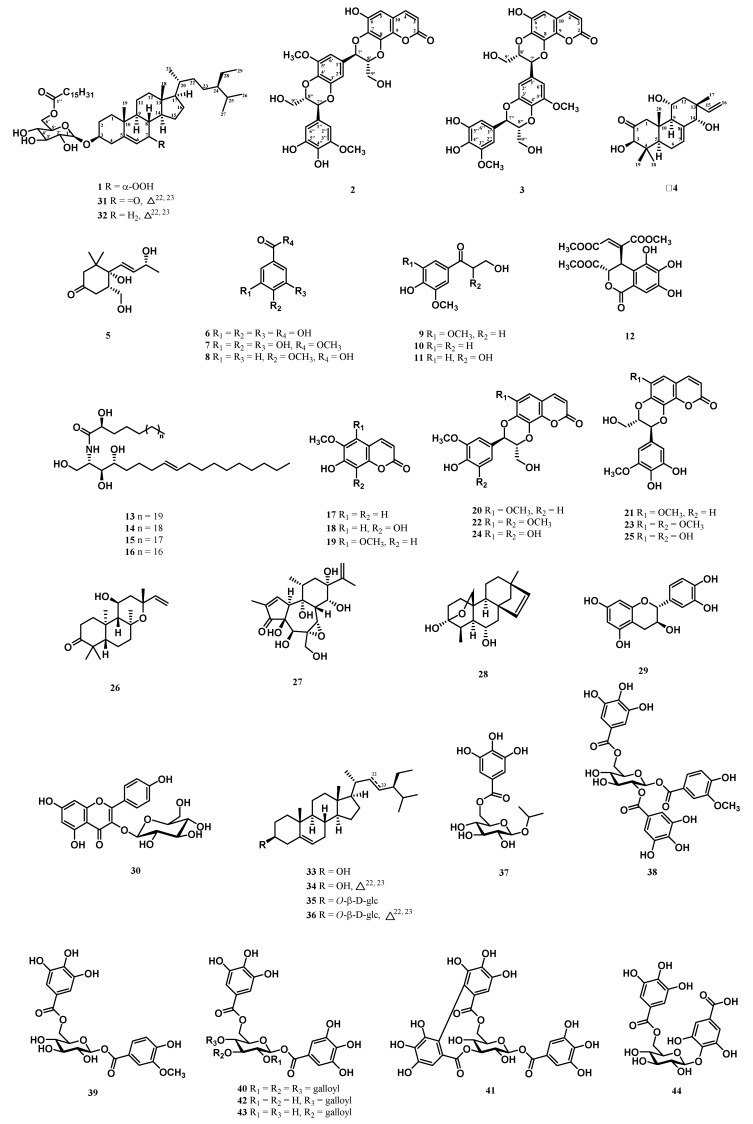
Structures of Compounds **1**–**44**.

Mangroves are distributed from tropical to subtropical regions of the world, comprising numerous bioactive compounds, which motivated scientists to explore further [12,13]. The *Excoecaria* genus (Euphorbiaceae family) comprises 37 species with acceptable names, distributed in the mangrove region of tropical Africa, Asia, and Australia [14]. The resin of *Excoecaria* have been used as a substitute for agarwood incense. Previous studies of *Excoecaria* species identified various classes of chemical constituents, such as diterpenes [15], flavonoids [16], galloyl glucoses [17], and triterpenes [18]. Numerous bioactivities of this genus have been shown as well, such as cytotoxic [19], anti-inflammatory [20], and anti-HIV [15] activity. *Excoecaria formosana* is a branched stout shrub mainly distributed in Tonkin, Indochina, the southern part of Taiwan, in thickets and forests along the seashores [21]. Previous investigation of the twigs of *Excoecaria formosana* led to the isolation of six diterpenes, and some compounds showed antimicrobial activities against *Bacillus subtilis* [22]. However, this species has never been reported on the chemopreventive constituents and other skeletons of compounds except for diterpenes. The stem and root of this plant have also never been investigated. Recently, we carried out a series of screenings and identified that the methanolic extracts of the stem and leaves of *E. formosana* exhibited GNMT-promoter-enhancing activity, and that the methanolic extracts of the leaves of *E. formosana* reduced the activity of NRF2 in Huh7 cells. After successfully investigating the whole plant of *E*. *formosana*, we isolated a new steroid, two new coumarinolignans, and a new diterpene, together with 40 known compounds (Figure 1). The 1D and 2D NMR spectroscopy techniques were used for structure elucidation and the identification of compounds [23], which have been applied in the chemical analysis of natural compounds for decades [24,25]. The 1D and 2D NMR spectra of new compounds are available in Supplementary Materials. In addition to GNMT-inducing activity, the effects of these compounds on NRF2 activity were also evaluated. The structure identification of new compounds and anti-HCC activity results are illustrated below.

## 2. Results

### 2.1. Structure Elucidation of 7α-Hydroperoxysitosterol-3-O-*β-d*-(6-O-palmitoyl)glucopyranoside (***1***)

**Figure 2:**
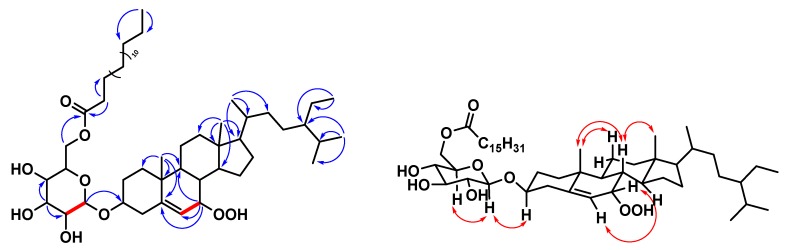
Key ^1^H-^1^H COSY (━), HMBC (H→C), and ROESY (H↔H) correlations of Compound **1**.

Compound **1** was obtained as a whitish solid with [α]^23^_D_ + 36 (*c* 0.13, CHCl_3_), and its molecular formula was deduced as C_51_H_90_O_9_ from HRESIMS data (*m/z* 869.64792 [M + Na]^+^ (calcd. C_51_H_90_O_9_Na, 869.64771)), implying seven degrees of unsaturation. Its IR spectrum displayed peaks at 3387 (OH group) and 1730 (carbonyl group) cm^-1^. The ^1^H and ^13^C NMR spectra of **1** (Table 1) were similar to those of (6′-*O*-palmitoyl)-sitosterol-3-*O*-β-d-glucopyranoside [26], except that an exchangeable hydroperoxy group at *δ*_H_ 7.67 (1H, s, OOH-7, D_2_O exchangeable) and an oxymethine proton [*δ*_H_ 4.16 (1H, br t, *J* = 4.6 Hz, H-7); *δ*_C_ 78.5 (C-7)] of Compound **1** replaced the methylene group of (6′-*O*-palmitoyl)-sitosterol-3-*O*-β-d-glucopyranoside. It was also supported by HMBC correlations of **1** from H-7 (*δ*_H_ 4.16) to C-5 (*δ*_C_ 148.2), C-6 (*δ*_C_ 120.4) and C-9 (*δ*_C_ 43.5). The relative configuration of OOH–C(7) was α–oriented, confirmed by the chemical shift of C-6 (*δ*_C_ 120.4) [27]. Comparing with (24*R*) 24-ethyl-7β-hydroperoxy-cholest-5-en-3β-ol and (24*R*) 24-ethyl-7α-hydroperoxy-cholest-5-en-3β-ol [27], the chemical shift of C-6 was (*δ*_C_ 126.0) in 7-β-stereoisomer, while the chemical shift of C-6 was (*δ*_C_ 119.9) in 7-α-stereoisomer. Thus, the structure of Compound **1** was further confirmed by COSY and ROESY data (Figure 2), and elucidated as 7α-hydroperoxysitosterol-3-*O*-β-d-(6-*O*-palmitoyl)glucopyranoside. The assignment of ^13^C NMR resonances was confirmed by DEPT, HSQC, and HMBC (Figure 2) techniques.

### 2.2. Structure Elucidation of Excoecoumarin A (***2***) and Excoecoumarin B (***3***)

**Figure 3:**
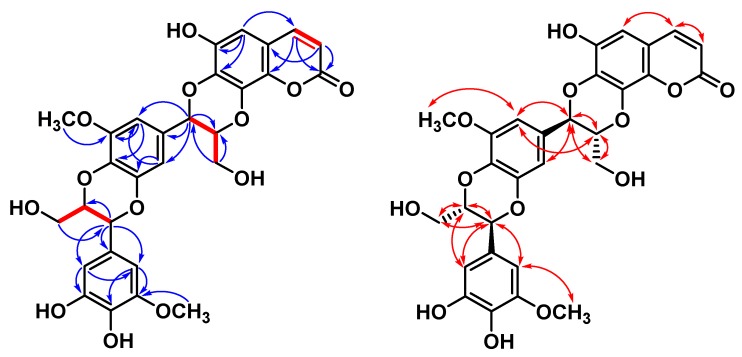
Key ^1^H-^1^H COSY (━), HMBC (H→C), and NOESY (H↔H) correlations of Compound **2**.

**Figure 4:**
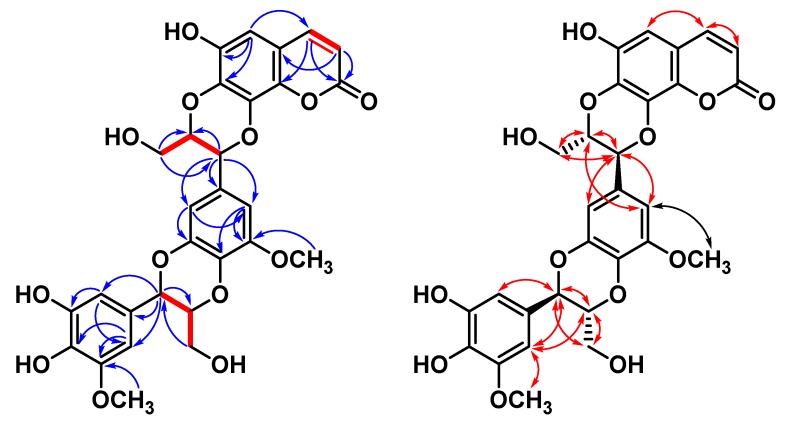
Key ^1^H-^1^H COSY (━), HMBC (H→C), and NOESY (H↔H) correlations of Compound **3**.

Excoecoumarin A (**2**) and excoecoumarin B (**3**) possessed the coumarinolignans generated by the formal condensation of coumarin with two additional 2-methoxy-3,4-dihydroxyphenyl-C_3_ moieties. They had almost identical spectroscopic properties (HRESIMS, ^1^H NMR, and ^13^C NMR) and physical data (appearance, UV, IR, and optical rotation), showing that they were very similar compounds. They were chromatographically separable as a light-yellowish amorphous powder with negative rotation. The positive-ion HRESIMS of Compounds **2** and **3** both established molecular formula C_29_H_26_O_13_Na showing a [M + Na]^+^ peak at *m/z* (**2**: 605.12642; **3**: 605.12633) (calcd. for C_29_H_26_O_13_Na, 605.12656). Maximal UV absorptions at **2**: 225 and 330 nm, and **3**: 230 and 330 nm, and IR absorption at **2**: 1702 cm^−1^, and **3**: 1699 cm^−1^ suggested the existence of coumarin residue. Taking the ^1^H NMR data of Compound **2** (Table 2), for example, the presence of two characteristic doublets was confirmed by the H-3 (*δ*_H_ 6.26) and H-4 (*δ*_H_ 7.79) with a coupling constant of 9.6 Hz. The chemical shift of H-4, being smaller than *δ*_H_ 7.8, suggested that there was no substituent at C-5 but a proton appearing at *δ*_H_ 6.62 (1H, s, H-5) [28]. HMBC correlations also showed a cross-peak between H-5 and C-4 (*δ*_C_ 146.4)/C-6 (*δ*_C_ 145.4)/C-7 (*δ*_C_ 138.5) (Figure 3 and Figure 4). A cross-peak between H-4 and H-5 in the NOESY experiment was also observed (Figure 3 and Figure 4). These signals were suggestive of the presence of a 6,7,8-trisubstituted coumarin moiety.

Subsequently, two of the 2-methoxy-3,4-dihydroxyphenyl-C_3_ moieties could be elucidated by NMR spectra. The ^1^H NMR spectrum showed proton signals for two AX patterns in the aromatic region [I: *δ*_H_ 6.77 (1H, d, *J* = 1.8 Hz, H-2′)/6.80 (1H, d, *J* = 1.8 Hz, H-6′); II: *δ*_H_ 6.57 (1H, d, *J* = 1.8 Hz, H-6″)/6.59 (1H, d, *J* = 1.8 Hz, H-2″; **2**)]. The ^1^H NMR spectrum further showed two aromatic methoxyl group at [I: *δ*_H_ 3.91 (3H, s, OCH_3_-5′); II: 3.85 (3H, s, OCH_3_-5″); **2**] along with two propanoid moiety attached to aromatic ring and dioxane at [I: *δ*_H_ 5.07 (1H, d, *J* = 7.8 Hz, H-7′), 4.21 (1H, ddd, *J* = 7.8, 4.1, 2.6 Hz, H-8′), 3.88 (1H, dd *J* = 12.6, 2.6 Hz, H-9′a), and 3.60 (1H, dd *J* = 12.6, 4.1 Hz, H-9′b); II: 4.84 (1H, d, *J* = 7.8 Hz, H-7″), 4.04 (1H, ddd, *J* = 7.8, 4.4, 2.6 Hz, 8″), 3.74 (1H, dd, *J* = 12.6, 2.6 Hz, H-9″a), and 3.54 (1H, dd, *J* = 12.6, 4.4 Hz, H-9″b); **2**] for H-7′, H-8′, and H-9, respectively. The presence of a 1,4-denzodioxane residue in these compounds was further supported by their ^13^C NMR spectra, showing typical signals at [I: *δ*_C_ 129.4 (C-1′), 110.6 (C-2′), 145.9 (C-3′), 135.2 (C-4′), 150.4 (C-5′), 105.52 (C-6′), 78.1 (C-7′), 79.9 (C-8′), and 61.8 (C-9′); II: *δ*_C_ 128.5 (C-1″), 104.1 (C-2″), 149.8 (C-3″), 136.0 (C-4″), 146.9 (C-5″), 109.4 (C-6″), 77.7 (C-7″), 80.1 (C-8″), and 62.1 (C-9″); **2**], respectively. In addition, the two oxymethines (H-7′/H-8′ and H-7′’/H-8″) were involved in ether linkages of benzene moiety, as determined by their chemical shifts, and were further confirmed with the HMBC spectrum (Figure 3 and Figure 4). The H-7′ showed ^2^*J* interactions with C-1′ and C-8′, and ^3^*J* correlations with C-2′ and C-6′. H-9′ correlated with C-7′ and C-8′. These correlations of the HMBC spectrum confirmed the attachment of hydroxymethyl at C-8′ and that of tetrasubstituted phenyl at C-7′. Additionally, H-7′’ showed ^2^*J* interactions with C-1″ and C-8″, and ^3^*J* correlations with C-2″ and C-6″. Hydroxymethyl H-9′ correlated with C-7″. These correlations of the HMBC spectrum confirmed the attachment of hydroxymethyl at C-8″ and that of tetrasubstituted phenyl at C-7″. The presence of methoxyl groups at C-5′ and C-3″ and hydroxyl groups at C-4″ and C-5″ could also be inferred through HMBC correlations. Aromatic proton H-2′ correlated with C-3′ and C-6′, and another aromatic proton H-6′ coupled with C-4′, C-5′, and C-7′. H-6″ showed interactions with C-5″, and another aromatic proton H-2′’ coupled with C-3″ and C-4″. Regarding the above NMR data, the linear condensation of 2-methoxy-3,4-dihydroxyphenyl-C_3_ (I)/2-methoxy-3,4-dihydroxyphenyl-C_3_ (II) residue was similar to that of signals of the literature compound, (*E*)-5-(3-(hydroxymethyl)-7-(3-hydroxyprop-1-enyl)-5-methoxy-2,3-dihydrobenzo[*b*][1,4]dioxin2-yl)-3-methoxybenzene-1,2-diol [29], except for the double bond on aliphatic side chain (3-hydroxyprop-1-enyl) of literature compound was reduced and formed 1,4-dioxane in **2** and **3**.

All spectroscopic data of individual Compounds **2** and **3** were explained by a diastereomeric character within the regioisomer pairs between stereochemical centers C-7′/C-8′. Fortunately, there existed a simple empirical pattern introduced by Merlini et al. [30]. In the regioisomeric benzodioxane, Compound **2**: C-7 was deshielded by a phenyl group on C-7′, and Compound **3**: C-7 was shielded by hydroxymethyl group on C-8′. Thus, the chemical shift of C-7 shifted to the high field when the connection converted from C-7-O—C-7′ (**2**) to C-7—O—C-8′ (**3**) [30,31]. Moreover, the *trans* disposition of H-7′/H-8′ and H-7′’/H-8′’ was confirmed by the coupling constant of H-7′ (*J* = 7.8 Hz) and H-7″ (*J* = 7.8 Hz) [32,33]. Accordingly, Compounds **2** and **3** were elucidated and named excoecoumarin A and excoecoumarin B, respectively.

From a biosynthetic point of view, the monomeric lignin precursors (**6**–**11**), coumarins (**17**–**19**), and the condensation of lignin with coumarins—coumarinolignans (**20**–**25**) were successively isolated from this plant. Therefore, we regarded Compounds **2** and **3** as natural plant constituents.

### 2.3. Structure Elucidation of Excoeterpenol A (***4***)

Compound **4** was assigned the molecular formula C_20_H_30_O_4_ through analysis of its HRESIMS (*m/z* 357.20352, [M + Na]^+^), requiring six degrees of unsaturation. Its IR spectrum showed absorptions at 3383, 1710, and 1578 cm^−1^, which were attributed to OH, C=O, and C=C functional groups. From the ^1^H and ^13^C NMR spectra (Table 3), one C=O group (*δ*_C_ 213.4), one C=C unit (*δ*_C_ 129.0, 138.6), and a vinyl C=CH_2_ group (*δ*_C_ 112.4, 147.2), which accounted for three of six degrees of unsaturation. Therefore, Compound **4** was proposed to be tricyclic.

The ^1^H NMR signals of Compound **4** (Table 3) showed four upfield tertiary methyl singlets (*δ*_H_ 0.80, 0.87, 1.00, 1.17), resonances for vinyl group of an ABX system (vinyl group) at *δ*_H_ 5.94 (1H, dd, *J* = 18.0, 11.1 Hz, H-15), 5.02 (1H, d, *J* = 18.0 Hz, H-16a), and 5.01 (1H, d, *J* = 11.1 Hz, H-16b), an additional olefinic H-atom at *δ*_H_ 5.83 (1H, dt, *J* = 6.6, 2.1 Hz, H-7), and three oxygenated CH group [*δ*_H_ 3.59 (1H, s, H-14), 3.82 (1H, ddd, *J* = 12.0, 10.5, 5.8 Hz, H-11), and 4.07 (1H, s, H-3) were observed. The remaining ^13^C-NMR (Table 3) and DEPT spectra showed signals corresponding to four methyl groups (*δ*_C_ 16.1, 17.1, 22.9, 29.2), three methylenes, five methines, and three quaternary carbons. The above observations and a comparison with the NMR data from closely related structures suggested that Compound **4** belonged to the isopimarane family of diterpenoids [34].

**Figure 5:**
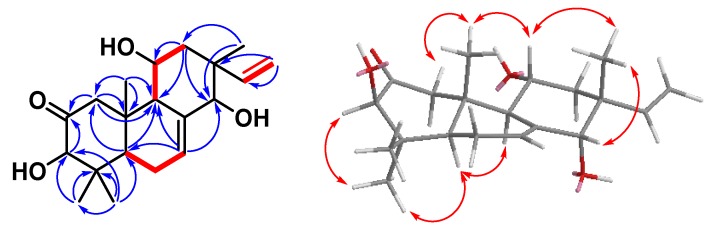
KEY ^1^H-^1^H COSY (━), HMBC (H→C), and NOESY (H↔H) correlations of Compound **4**.

The structure of Compound **4** showed close similarity to those of the literature compound, (3β,12α,13α)-3,12-dihydroxypimara-7,15-dien-2-one [34] after comparing their ^1^H and ^13^C NMR spectra. They differed only in the absence of the OH group at C-12 of the literature compound and the appearance of two additional OH signals at C-11 and C-14 in Compound **4**. Three significant differences in the chemical shift values of C-11, C-12, and C-14 in the literature compound allowed the two OH groups to be placed at C-11 and C-14 in Compound **4**. The HMBC spectrum (Figure 5) also confirmed it by correlations between H-12/C-11 and C-14, and between H-7 and H-17/C-14. The correlations of H-9↔H-11↔H-12, and H-5↔H-6↔H-7 were also observed in the COSY experiment (Figure 5) and further confirmed the structure of Compound **4**.

The relative configuration of Compound **4** was derived by a NOESY spectrum (Figure 5) in combination with biogenetic considerations and in comparison with the isopimarane family [35]. According to the NOESY spectrum, H-3 was assigned as α-oriented, which was confirmed by the 1,3-diaxil NOE H-3/H-1α. NOEs for H-3/H-18, H-18/H-5, and H-5/H-9 indicated that H-3, H-5, H-9, and H-18 were on the same side of the molecular plane, tentatively assumed as α-orientation. On the other hand, NOE cross-peaks H-20/H-11, H-11/H-17, and H-17/H-14 demonstrated the *cis*-β-orientation of protons at H-11, H-14, H-17, and H-20. On the basis of this evidence, the structure of Compound **4** was thus defined as (3β,11α,14α)-3,11,14-trihydroxypimara-7,15-dien-2-one and given the trivial name excoeterpenol A.

### 2.4. Identification of Known Compounds ***5**–**44***

By comparison of experiment and reported spectroscopic data ([α]_D_, UV, IR, NMR, and MS), known compounds were identified as one apocarotenoid: deglucosyl lauroside B (**5**) [36], seven benzenoids: gallic acid (**6**) [37], methyl gallate (**7**) [38], 4-methoxybenzoic acid (**8**) [39], 3-hydroxy-1-(3,5-dimethoxy-4-hydroxyphenyl)propan-1-one (**9**) [40], 3-hydroxy-1-(4-hydroxy-3-methoxyphenyl)propan-1-one (**10**) [41], 2,3-dihydroxy-1-(4-hydroxy-3-methoxyphenyl)propan-1-one (**11**) [41], and (2*S*,3*R*)-4*E*-dehydrochebulic acid trimethyl ester (**12**) [42], a mixture of cerebrosides: gynuramides I–IV (**13**–**16**) [43], three coumarins: scopoletin (**17**) [44], fraxetin (**18**) [45], and 6-hydroxy-5,7-dimethoxycoumarin (**19**) [46], six coumarinolignans: cleomiscosins A–D (**20**–**23**) [47,48,49], malloapelin A (**24**) [32,33], and malloapelin B (**25**) [32,33], three diterpenes: *ent*-11-α-hydroxy-3-oxo-13-*epi*-manoyl oxide (**26**) [50], excoecafolin D (**27**) [15], and agallochin I (**28**) [51], two flavonoids: (+)-catechin (**29**) [52] and kaempferol-3-*O*-β-d-glucoside (**30**) [53], six steroids: 6′-(stigmast-5-en-7-one-3-*O*-β-glucopyransidyl)hexadecanoate (**31**) [54], (6′-*O*-palmitoyl)sitosterol-3-*O*-β-d-glucoside (**32**) [26], a mixture of β-sitosterol (**33**) and stigmasterol (**34**) [55], a mixture of 3-*O*-β-d-glucopyranosyl β-sitosterol (**35**) and 3-*O*-β-d-glucopyranosyl stigmasterol (**36**) [56], and eight galloyl glucoses: isopropyl *O*-β-(6′-*O*-galloyl)glucopyranoside (**37**) [57], 4-hydroxy-3-methoxyphenol 1-*O*-β-d-(2′,6′-di-*O*-galloyl)glucoside (**38**) [58], 3-methoxy-4-hydroxyphenyl 1-*O*-β-d-(6′-*O*-galloyl)glucopyranoside (**39**) [59], 1,2,3,4,6-penta-*O*-galloyl-β-d-glucose (**40**) [60], corilagin (**41**) [61], 1,4,6-tri-*O*-galloyl-β-d-glucose (**42**) [62], 1,3,6-tri-*O*-galloyl-β-d-glucose (**43**) [63], and gallic acid 4-*O*-β-d-(6′-*O*-galloyl)-glucose (**44**) [64]. The phytochemical data of known compounds are available in Supplementary Materials.

### 2.5. Bioactivity Results

**Figure 6:**
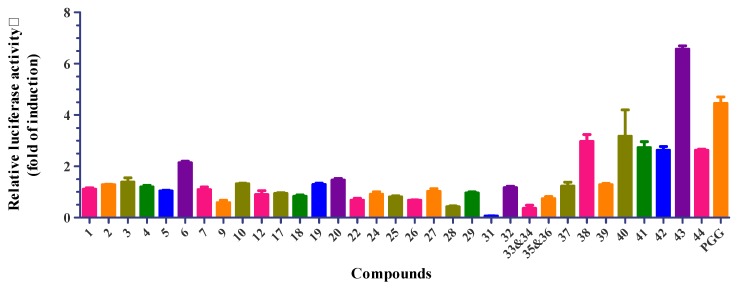
Glycine *N*-methyltransferase (GNMT)-promoter-enhancing activity (fold of induction) of compounds from the whole plant of *E. formosana.* Sample concentration was 100 µM. GNMT activity (fold of induction) = observed activity/solvent control activity; 1,2,3,4,6-penta-*O*-galloyl-β-d-glucose (PGG) was used as positive control for GNMT activation with 100 µM.

**Figure 7:**
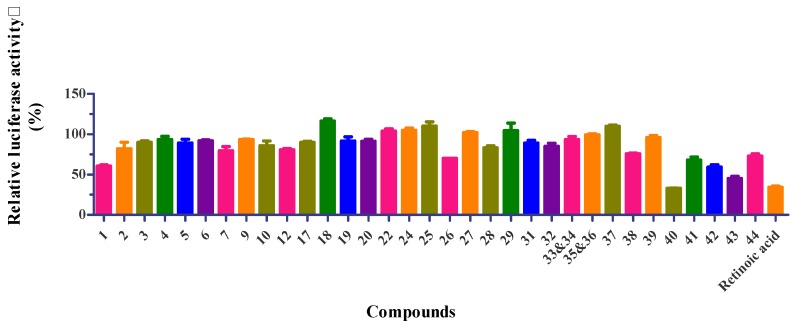
Nuclear factor erythroid 2-related factor 2 (NRF2) inhibition in Huh7 cells of compounds from the whole plant of *E. formosana*. Sample concentration was 100 µM. Relative NRF2 activity presented as percentage to solvent control. Retinoic acid is used as positive control for NRF2 inhibition with 1 µM.

Thirty-five isolates (Compounds **1**‒**7**, **9**, **10**, **12**, **17**‒**20**, **22**, **24**‒**29**, and **31**‒**44**) were evaluated for their GNMT-promoter-enhancing activity (Figure 6), and suppressive effects on NRF2 activity in Huh7 cells (Figure 7). The numerical value of bioactivity results, see Supplementary Materials. Gallotannins (Compounds **38**, **40**, **42**‒**44**) and ellagitannin (Compound **41**) showed GNMT-promoter-enhancing activity in 100 μM concentrations, with a 2.97 ± 0.27-, 3.17 ± 1.03-, 2.73 ± 0.23-, 2.63 ± 0.14-, 6.57 ± 0.13-, and 2.62 ± 0.05-fold increase, respectively. Furthermore, only Compounds **40** and **43** exhibited NRF2-inhibitory effects in the Huh7 cells with a related activity of 33.1 ± 0.2% and 45.2 ± 2.5%.

## 3. Discussion

Previously, Kant and his colleagues reported that 1,2,3,4,6-penta-*O*-galloyl-β-d-glucose (PGG) is a glycine *N*-methyltransferase (GNMT) inducer. They showed that PGG could not only induce apoptosis in Huh7 cells, but also sensitize Huh7 cells to sorafenib treatment [8]. Since PGG is made of a glucose core with five galloyl groups, a few interesting questions remain unanswered: whether five galloyl groups are required for GNMT-promoter-enhancing activity, and, if not, whether the substituted position of galloyl groups is critical for the activity. The structure-activity-relationship (SAR) study depicted that more than two galloyl groups connected with the glucose core were necessary for GNMT-promoter-enhancing activity. As isopropyl *O*-β-(6′-*O*-galloyl)glucopyranoside (**37**) and 3-methoxy-4-hydroxyphenyl 1-*O*-β-d-(6′-*O*-galloyl)glucopyranoside (**39**) only had one galloyl group connected with the glucose core, they did not show GNMT-promoter-enhancing activity. Gallic acid 4-*O*-β-d-(6′-*O*-galloyl)-glucose (**44**) is an unusual galloyl glucose compound with one ester linkage group and one ether linkage galloyl group connected with the glucose core. Surprisingly, Compound **44** also depicted GNMT-promoter-enhancing activity. Further evidence is required to support whether the numbers of the ether linkage galloyl group or the substituted position of the galloyl group on the glucose core affect GNMT-promoter-enhancing activity. Compounds with three galloyl groups connected with the glucose core, including gallotannins (Compounds **38**, **40**, **42**, and **43**) and ellagitannin (Compound **41**) showed GNMT-promoter-enhancing activity. Among them, 1,3,6-tri-*O*-linkage (**43**) was the most active compound, two-fold higher in induction activity than 1,4,6-tri-*O*-linkage (**42**) and positive control PGG. This finding suggested that the substituted position of galloyl groups is crucial for GNMT-promoter-enhancing activity. Gallotannin **43** showed better GNMT-promoter-enhancing activity than ellagitannin **41**, suggesting different types of galloyl glucose change their GNMT-promoter-enhancing activity. Previous biological studies of tannin analogs were found to have antiviral, antibacterial, antioxidant, and antitumor activity [65,66]. Surprisingly, this is the first time that the SAR of tannin analogs is discussed with GNMT-promoter-enhancing activity. More importantly, our results suggested that three galloyl groups at the 1,3,6 position as gallotannin were the best GNMT inducer.

Transcriptional factor NRF2, a master regulator for antioxidative and detoxification responses, is implicated in chemoresistance in several cancers [67]. As mentioned, PGG has been shown to sensitize HCC cells to sorafenib treatment. Thus, it is interesting to test whether these gallotannins can inhibit NRF2 activity in HCC cells. Surprisingly, we found that Compounds **40** and **43,** the two most potent GNMT inducers, also suppressed NRF2 activity in Huh7 cells. These results indicated that the sensitization ability of PGG could be attributed to its inhibitory effect on NRF2 activity. However, further investigations are needed to delineate the underlying mechanisms.

Two new coumarinolignans, excoecoumarins A and B (Compounds **2** and **3**), with an unprecedented carbon skeleton, together with two new compounds, were obtained from the whole plant of *E. formosana*, and their structures were established by spectroscopic analysis. Coumarinolignans have many interesting biological activities, and their cytotoxic activity and hepatoprotective potential are globally known. Although the coumarinolignans in this study did not show biological effects on GNMT-promoter and NRF2 activity, this indicated that the coumarinolignans might exert its activity via mechanisms not involving these two proteins. To our knowledge, this is the first report on the GNMT-promoter-enhancing and NRF2 suppressing activities of this plant in Huh 7 cells. Further evidence is needed to support whether *E. formosana* is helpful to HCC patients.

## 4. Materials and Methods

### 4.1. General Experiment Procedures

All melting points were determined on a Yanaco micromelting apparatus (Yanaco, Kyoto, Japan) and were uncorrected. Optical rotations were measured on a Jasco P-2000 polarimeter (Jasco, Kyoto, Japan), UV spectra were obtained with a Jasco-V-530 UV/vis spectrophotometer (Jasco, Kyoto, Japan), and IR spectra (ATR) were acquired with a Jasco FT/IR-4600 spectrometer. We recorded 1D (^1^H, ^13^C, DEPT) and 2D (COSY, NOESY, ROESY, HSQC, HMBC) NMR spectra on a Varian Germini-2000 spectrometer (Varian, Inc. Vacuum Technologies, Lexington, MA, USA) operated at 200 (1H) and 50 MHz (^13^C), Varian Unityplus-400 spectrometer (Varian, Inc. Vacuum Technologies, Lexington, MA, USA) operated at 400 (^1^H) and 100 MHz (^13^C), Varian Mercuryplus-400 spectrometer (Varian, Inc. Vacuum Technologies, Lexington, MA, USA) operated at 400 (^1^H) and 100 MHz (^13^C), and Varian VNMRS-600 spectrometer (Varian, Inc. Vacuum Technologies, Lexington, MA, USA) operated at 600 (^1^H) and 150 MHz (^13^C). Low-resolution mass spectra were obtained with POLARIS Q Thermo Finnigan (Thermo Fisher Scientific, Chicago, IL, USA), Water ZQ 4000 (Waters, Milford, MA, USA), and VG Quattro GC/MS/MS/DS (Waters, Milford, MA, USA) mass spectrometers. HRESIMS were recorded on a Bruker APEX II mass spectrometer (Bruker, Karlsruhe, Germany). Silica gel (70–230 and 230–400 mesh; Silicycle, Quebec, Canada) was used for column chromatography (CC), and silica gel 60 F254 (Merck, Darmstadt, Germany) and RP-18 F254S (Merck, Darmstadt, Germany) were used for TLC and preparative TLC, respectively, visualized with a Ce_2_(SO4)_3_ aqueous solution. Further purification was performed by medium-performance liquid chromatography (MPLC; ceramic pump: VSP-3050; EYELA, Kyoto, Japan).

### 4.2. Plant Material

The whole plant of *E. formosana* was collected in February 2015 at Mudan, Pingtung county, Taiwan, and identified by I.-S. C. A voucher specimen (Chen 5626) was deposited with the herbarium of the College of Pharmacy, Kaohsiung Medical University, Kaohsiung, Taiwan.

### 4.3. Extraction and Isolation

The dried whole plant (leaves, stem, and root; 5.7 kg) of *E. formosana* was extracted at room temperature with MeOH (30 L) three times to yield an MeOH extract (465 g) that was partitioned between EtOAc and H_2_O (1:1) to provide an EtOAc-soluble (110 g) and an H_2_O-soluble fraction. The H_2_O-soluble fraction was then partitioned between *n*-butanol and H_2_O (1:1) to obtain an *n*-butanol-soluble (100 g) and H_2_O-soluble layer (255 g). The EtOAc-soluble fraction (100 g) was subjected to column chromatography (silica gel; column size: 10 cm × 80 cm; *n*-hexane/EtOAc 100:1 to 100% acetone, then finally washed with 100% methanol) to yield 12 subfractions (Fr. 1–12). Fr. 5 (18 g) was recrystallized from MeOH to give Compounds **33** and **34** (233 mg; R*_f_* = 0.48 in *n*-hexane/acetone 4/1). Fr. 9 (2.7 g) was subjected to MPLC (silica gel; *n*-hexane/ acetone 4:1; column size: 3 cm × 30 cm) to give 12 fractions (Fr. 9-1–9-12). Fr. 9-5 was subjected to MPLC (silica gel; CH_2_Cl_2_/acetone 40:1; column size: 1.5 cm × 30 cm) to afford ten fractions (Fr. 9-5-1–9-5-10). Fr. 9-5-7 was further separated with prep. TLC (CH_2_Cl_2_/EtOAc = 40:1) to obtain Compound **26** (2.0 mg; R*_f_* = 0.35). Fr. 9-11 was subjected to MPLC (silica gel; CH_2_Cl_2_/acetone 2:1; column size: 2.5 cm × 30 cm) to give ten fractions (Fr. 9-11-1–9-11-10). Fr. 9-11-8 was subjected to MPLC (silica gel; *n*-hexane/CH_2_Cl_2_/methanol 4/20/1; column size: 2 cm × 30 cm) to generate eight fractions (Fr. 9-11-8-1–9-11-8-8). Fr. 9-11-8-2 was subjected to MPLC (silica gel; CH_2_Cl_2_/EtOAc/methanol 50/10/2; column size: 2 cm × 30 cm) to furnish Compound **31** (19.5 mg; R*_f_* = 0.48). Fr. 9-11-8-3 was subjected to MPLC (silica gel; CH_2_Cl_2_/methanol 25:1; column size: 2 cm × 30 cm) to yield four fractions (Fr. 9-11-8-3-1–9-11-8-3-4). Fr. 9-11-8-3-2 was subjected to MPLC (RP-18; methanol/acetone 1:1; column size: 1.5 cm × 30 cm) to provide eight fractions (Fr. 9-11-8-3-2-1–9-11-8-3-2-8). Fr. 9-11-8-3-2-3 was subjected to MPLC (silica gel; *n*-hexane/CH_2_Cl_2_/methanol 40/20/1; column size: 1.5 cm × 30 cm) to afford Compounds **1** (2.6 mg; R*_f_* = 0.42) and **32** (5.8 mg; R*_f_* = 0.38). Fr. 9-11-3 was subjected to MPLC (silica gel; CH_2_Cl_2_/methanol 16/1; column size: 2 cm × 30 cm) to give nine fractions (Fr.9-11-3-1–9-11-3-9). Fr. 9-11-3-7 was subjected to MPLC (RP-18; H_2_O/acetone 1.5/1; column size: 2 cm × 30 cm) to obtain Compound **7** (46.8 mg; R*_f_* = 0.45). Fr. 10 was subjected to subjected to silica gel column chromatography (column size: 5.5 cm × 70 cm) and eluted with CH_2_Cl_2_, EtOAc, and methanol, to which EtOAc and MeOH were gradually added to increase the polarity resulting in ten fractions (Fr. 10-1–10-10). Fr. 10-7 was subjected to MPLC (RP-18; H_2_O/MeOH 1/1; column size: 2 cm × 30 cm) to give 11 fractions (Fr. 10-7-1–10-7-11). Fr.10-7-1 was separated with Sephadex LH-20 (column size: 1.5 cm × 45 cm) and eluted with MeOH to furnish Compounds **10** (2.0 mg; R*_f_* = 0.5 in H_2_O/methanol 1/1) and **29** (9.6 mg; R*_f_* = 0.73 in H_2_O/methanol 1/1). Fr. 10-7-10 was subjected to MPLC (RP-18; H_2_O/acetone 1/2; column size: 2 cm × 30 cm) to yield 26 fractions (Fr. 10-7-10-1–10-7-10-26). Fr.10-7-10-5 was subjected to MPLC (silica gel, CH_2_Cl_2_/EtOAc 3:2; column size: 1.5 cm × 30 cm) to gain Compound **28** (4.3 mg; R*_f_* = 0.13). Fr. 10-8 was subjected to MPLC (silica gel, CH_2_Cl_2_/methanol 20:1; column size: 2.5 cm × 30 cm) to gain 10 fractions (Fr. 10-8-1–10-8-10). Fr. 10-8-4 was subjected to MPLC (RP-18; H_2_O/acetone 2/1; column size: 2 cm × 30 cm) to obtain Compound **11** (1.5 mg; R*_f_* = 0.38). Fr. 10-8-4-9 was subjected to MPLC (RP-18; H_2_O/acetone 1/1; column size: 1.5 cm × 30 cm) to obtain Compound **4** (1.3 mg; R*_f_* = 0.25). Fr.10-4 was subjected to MPLC (silica gel, *n*-hexane/acetone 4:1; column size: 2 cm × 30 cm) to give ten fractions (Fr. 10-4-1–10-4-10). Fr.10-4-10 was subjected to MPLC (silica gel, CH_2_Cl_2_/acetone 30:1; column size: 1.5 cm × 30 cm) and separated with prep. TLC (CH_2_Cl_2_/acetone 30:1) to obtain Compound **19** (3.2 mg; R*_f_* = 0.25). Separation of fraction 11 on a silica gel column (from CH_2_Cl_2_/methanol 60:1 to methanol 100%; column size: 8.5 cm × 70 cm) furnished 22 subfractions (Fr. 11-1–11-22). Fr. 11-12 was recrystallized from MeOH to give a mixture of Compounds **13**–**16** (15 mg; R*_f_* = 0.5 in CH_2_Cl_2_/methanol 6/1). Fr. 11-8 was subjected to MPLC (RP-18; H_2_O/acetone 1/2; column size: 2 cm × 30 cm) to generate 11 fractions (Fr. 11-8-1–11-8-11). Fr. 11-8-1 was separated with prep. TLC (CH_2_Cl_2_/acetone 8:1) to gain Compound **9** (3.7 mg; R*_f_* = 0.25). Fr. 11-8-2 was subjected to MPLC (silica gel; CH_2_Cl_2_/acetone 10/1; column size: 2 cm × 30 cm) to afford three fractions (Fr. 11-8-2-1–11-8-2-3). Fr. 11-8-2-1 was separated with prep. TLC (CH_2_Cl_2_/acetone 8:1) to obtain Compound **17** (1.7 mg; R*_f_* = 0.65). Fr. 11-8-3 was subjected to MPLC (RP-18; H_2_O/acetonitrile 2.5/1; column size: 1.5 cm × 30 cm) to produce Compound **8** (0.9 mg; R*_f_* = 0.3). Fr. 11-8-2-2 was recrystallized and washed by acetone, and the washing liquor (45.2 mg) was subjected to MPLC (RP-18; H_2_O/acetonitrile 2.5/1; column size: 1.5 cm × 30 cm) to generate nine fractions (Fr. 11-8-2-2-1–11-8-2-2-9). Fr. 11-8-2-2-M-5 was separated with prep. TLC (CH_2_Cl_2_/acetone 8/1) to obtain Compound **23** (3.7 mg; R*_f_* = 0.3). Fr. 11-8-2-2-C was separated with prep. TLC (CH_2_Cl_2_/acetone 8:1) to gain Compound **22** (3.7 mg; R*_f_* = 0.3). Fr. 11-8-2-2-C-A was subjected to MPLC (silica gel; benzene/EtOAc 1:1; column size: 1.5 cm × 30 cm) to give Compounds **20** (1.6 mg; R*_f_* = 0.3) and **21** (1.8 mg; R*_f_* = 0.25). Fr. 11-15 was subjected to MPLC (RP-18; H_2_O/acetone 2.5/1; column size: 2.5 cm × 30 cm) to generate 16 fractions (Fr. 11-15-1–11-15-16). Fr. 11-15-8 was subjected to MPLC (RP-18; H_2_O/MeOH 1.5/1; column size: 2 cm × 30 cm) to give nine fractions (Fr. 11-15-8-1–11-15-8-9). Fr. 11-15-8-3 was subjected to MPLC (CHCl_3_/MeOH 14/1; column size: 1.5 cm × 30 cm) to obtain Compound **25** (38 mg; R*_f_* = 0.13). Fr. 11-15-8-3-2 was purified by MPLC (silica gel, benzene/acetone 2:1; column size: 1.5 cm × 30 cm) to furnish Compound **24** (3.9 mg; R*_f_* = 0.25). Fr. 11-15-10 was subjected to MPLC (RP-18; H_2_O/acetone 2/1; column size: 1.5 cm × 30 cm) to give six fractions (Fr. 11-15-10-1–11-15-10-6). Fr. 11-15-10-3 was subjected to MPLC (RP-18; H_2_O/acetonitrile 2/1; column size: 1.5 cm × 30 cm) to afford 5 fractions. Fr.11-15-10-3-4 was subjected to HPLC (biphenyl, W/M 1:1.5, flow = 2) to obtain Compounds **2** (9.2 mg; R*_f_* = 0.5) and **3** (8.3 mg; R*_f_* = 0.43). Fr. 11-15-5 was subjected to MPLC (silica gel; CH_2_Cl_2_/acetone 1/1; column size: 1.5 cm × 30 cm) to give eight fractions (Fr. 11-15-5-1–11-15-5-8). Fr. 11-15-5-3 was subjected to MPLC (silica gel, benzene/acetone 1.5:1; column size: 1.5 cm × 30 cm) to gain Compound **27** (7.7 mg; R*_f_* = 0.25). Fr. 11-15-16 was recrystallized from CH_2_Cl_2_-MeOH to obtain Compounds **35** and **36** (110 mg; R*_f_* = 0.48 in H_2_O/acetone 2/1). Fr. 11-13 was subjected to MPLC (RP-18; H_2_O/MeOH 3/2; column size: 2 cm × 30 cm) to afford five fractions (Fr. 11-13-1–11-13-5). Fr. 11-13-1 was subjected to Sephadex LH-20 (column size: 2 cm × 45 cm) and eluted with MeOH to give 11 fractions (Fr. 11-13-1-1– 11-13-1-11). Fr. 11-13-1-2 was separated by MPLC (RP-18; H_2_O/MeOH 2/1; column size: 1.5 cm × 30 cm) to obtain Compound **5** (3.2 mg; R*_f_* = 0.25). Fr. 11-13-1-4 was separated with prep. TLC (RP-18; H_2_O/MeOH 1/1) to furnish four fractions (Fr. 11-13-1-4-1–11-13-1-4-4). Fr. 11-13-1-4-A was subjected to HPLC (RP-18; H_2_O/MeOH 1/1) to give Compound **12** (2.1 mg; R*_f_* = 0.5). Fr. 11-13-1-6 was subjected to MPLC (RP-18; H_2_O/MeOH 2/1; column size: 1.5 cm × 30 cm) to produce two fractions (Fr. 11-13-1-6-1–11-13-1-6-2). Fr. 11-13-1-6-2 was separated with prep. TLC (RP-18; H_2_O/MeOH 1/1) to obtain Compound **18** (5.6 mg; R*_f_* = 0.58). Fr. 11-20 was separated with Sephadex LH-20 (column size: 3 cm × 45 cm) and eluted with MeOH to provide 16 fractions. Fr.11-20-7 was subjected to MPLC (RP-18; H_2_O/MeOH 2/1; column size: 2 cm × 30 cm) to afford Compound **37** (5.0 mg; R*_f_* = 0.3). Fr. 11-20-13 was subjected to MPLC (RP-18; H_2_O/MeOH 2/1; column size: 2 cm × 30 cm) to obtain Compound **38** (2.4 mg; R*_f_* = 0.38). Fr. 11-20-13-13 was subjected to MPLC (RP-18; H_2_O/acetone 3/2; column size: 1.5 cm × 30 cm) to give 2 fractions (Fr. 11-20-13-13-1–11-20-13-13-2). Fr. 11-20-13-13-2 was separated with HPLC (RP-18; W/M 1:1.5) to furnish Compound **30** (0.7 mg; R*_f_* = 0.5). Fr. 11-20-10 was subjected to MPLC (RP-18; H_2_O/MeOH 4/1; column size: 2 cm × 30 cm) to generate Compound **39** (14.2 mg; R*_f_* = 0.75). Fr. 11-21 and 22 were separated with Sephadex LH-20 (column size: 3 cm × 45 cm) and eluted with MeOH to afford 11 fractions (Fr. 11-21-1–11-21-11). Fr. 11-21-11 was subjected to MPLC (RP-18, H_2_O/MeOH 2:1; column size: 2 cm × 30 cm) to gain Compound **40** (13.8 mg; R*_f_* = 0.33). Fr. 11-21-8 was subjected to MPLC (RP-18, H_2_O/MeOH 3:1; column size: 2 cm × 30 cm) to generate Compound **42** (13.8 mg; R*_f_* = 0.25). Fr. 11-21-8-5 was subjected to Sephadex LH-20 (column size: 2 cm × 45 cm) and eluted with H_2_O/EtOH 1:1 (column size: 2 cm × 45 cm) to give Compound **43** (11.5 mg; R*_f_* = 0.48 in H_2_O/MeOH 3/1). Fr. 11-21-3 was subjected to Sephadex LH-20 (column size: 2 cm × 45 cm) and eluted with H_2_O /EtOH 1:1 (column size: 2 cm × 45 cm) to afford 7 fractions (Fr. 11-21-3-1–11-21-3-7). Fr. 11-21-3-6 was subjected to MPLC (RP-18, W/MeOH 3:1; column size: 1.5 cm × 30 cm) to obtain Compound **6** (6.8 mg; R*_f_* = 0.83). Fr. 11-21-4 was subjected to MPLC (RP-18; H_2_O/MeOH 4/1; column size: 2 cm × 30 cm) to produce Compound **44** (2.9 mg; R*_f_* = 0.45). Fr. 12 was subjected to a Diaion HP-20 (column size: 2 cm × 45 cm) and eluted with MeOH-H_2_O (50%, 4000 mL), (75%, 4000 mL), and methanol 400 mL to furnish three fractions (Fr. 12-1–12-3). Fr. 12-1 was subjected to Sephadex LH-20 (column size: 2 cm × 45 cm) and eluted with MeOH to generate Compound **41** (260 mg; R*_f_* = 0.58 in H_2_O/MeOH 3/1).

### 4.4. 7-Hydroperoxysitosterol-3-O-*β-d*-(6-O-palmitoyl)glucopyranoside (***1***)

Whitish solid; [α] ^23^_D_ + 36 (*c* 0.13, CHCl_3_), IR *υ*_max_ (ATR) 3387 (OH), 1730 (C=O) cm^−1^; ^1^H NMR and ^13^C NMR (Table 1); ESIMS *m/z*: 869 [M + Na]^+^; HRESIMS *m/z*: 869.64792 [M + Na]^+^, calcd.: C_51_H_90_O_9_Na, 869.64771.

### 4.5. Excoecoumarin A (***2***)

Light-yellowish amorphous powder; [α]^25^_D_: –218 (*c* 0.1, MeOH); UV (MeOH) λ_max_ (log *ε*) 220 (4.68), 320 (3.96) nm; UV (MeOH + KOH) λ_max_ (log *ε*) 225 (4.68), 330 (3.91) nm; IR *υ*_max_ (ATR) cm^−1^: 3404 (OH), 1702 (C=O), 1606, 1575, 1514 (aromatic ring); ^1^H NMR and ^13^C NMR (Table 2); ESIMS *m/z* 583 [M + H]^+^; HRESIMS *m/z*: 605.12642 [M + Na]^+^, calcd.: C_29_H_26_O_13_Na, 605.12656.

### 4.6. Excoecoumarin B (***3***)

Light-yellowish powder; [α]^25^_D_: –254 (*c* 0.075, MeOH); UV (MeOH) λ_max_ (log *ε*) 220 (4.76), 320 (4.07) nm; UV (MeOH + KOH) λ_max_ (log *ε*) 230 (4.64), 330 (4.02) nm; IR *υ*_max_ (ATR) cm^−1^: 3464 (OH), 1699 (C=O), 1608, 1575, 1514 (aromatic ring); ^1^H NMR and ^13^C NMR (Table 2); ESIMS *m/z* 583 [M + H]^+^; HRESIMS *m/z*: 605.12633 [M + Na]^+^, calcd.: C_29_H_26_O_13_Na, 605.12656.

### 4.7. Excoeterpenol A (***4***)

Whitish solid; [α]^25^_D_: +14 (*c* 0.065, MeOH); IR *υ*_max_ (ATR): 3383 (OH), 1710 (C=O), 1578 (C=C) cm^−1^; ^1^H NMR and ^13^C NMR (Table 3); ESIMS *m/z* 335 [M + H]^+^; HRESIMS *m/z*: 357.20352 [M + Na]^+^, calcd.: C_20_H_30_O_4_Na, 357.20363.

### 4.8. Cell Culture

Huh7 cell stable expression GNMT-promoter reporter [8] and NRF2 reporter [68] were cultured in Dulbecco’s Modified Eagle’s Medium (DMEM) (Gibco BRL, Grand Island, NY, USA) with 10% heat-inactivated fetal bovine serum (HyClone, Logan, UT, USA), penicillin (100 U/mL), and streptomycin (100 μg/mL) supplemented with 1 μg/mL puromycin and 100 μg/mL hygromycin, respectively, in a humidified incubator with 5% CO_2_.

### 4.9. Luciferase Reporter Assay

Reporter cells were seeded (1 × 104 cells/well) in 96-well plate, then treated with indicated concentrations of compounds for 18 h. At the assay time point, resazurin (Cayman Chemical, Ann Arbor, MI, USA) was added to a final concentration of 0.1 mg/mL and further incubated for 4 h at 37 °C. Fluorescence of the reduced resazurin (ex/em: 530/590 nm) was measured from the culture supernatant by using a Synergy HT Multi-Mode Reader (BioTek, Winooski, VT, USA) to determine cell viability. The cells were then harvested for luciferase activity measurements according to the manufacturer’s protocol (Promega Corporation, Madison, WI, USA). Relative luciferase activity was calculated by normalizing luciferase activity to cell viability. For GNMT promoter activity assay treatment, data were presented as the fold to DMSO solvent control; for NRF2-activity assay, DMSO solvent control was used as 100% activity.

## Figures and Tables

**Table 1:** ^1^H (600 MHz, CDCl_3_) and ^13^C NMR (150 MHz, CDCl_3_) data of Compound **1**.

Position	1	
	*δ*_C_	*δ*_H_ (m, *J* in Hz)
1	37.6	
2	28.2	
3	79.2	3.65, m
4	39.0	
5	148.2	
6	120.4	5.75 (dd, 4.6, 1.8)
7	78.5	4.16 (br t, 4.6)
8	45.8	
9	43.5	
10	36.8	
11	22.7	
12	39.0	
13	42.3	
14	49.0	
15	20.9	
16	25.9	
17	55.6	
18	11.3	0.66, s
19	18.2	0.99, s
20	36.1	
21	18.8	0.92 (d, 6.6)
22	33.9	
23	25.9	
24	45.8	
25	29.3	
26	19.8	0.81 (d, 6.2)
27	19.0	0.82 (d, 6.2)
28	23.1	
29	12.0	0.88 (t, 7.2)
1′	101.4	4.39 (d, 8.1)
2′	73.5	3.36, m
3′	75.9	3.58 (t, 9.0)
4′	70.0	3.38, m
5′	74.0	3.47, m
6′	63.0	4.26 (d, 11.7) 4.50 (dd, 11.7, 4.8)
1″	174.9	
2″	34.2	2.36 (t, 7.8)
3″	24.9	
4″–13″	29.4–29.7 *^a^*	
14″	31.9	
15″	22.7	
16″	14.1	0.84 (t, 6.3)
OOH-7		7.67, s, D_2_O exchangeable

*^a^* Overlapped signals reported without designating multiplicity.

**Table 2:** ^1^H (600 MHz, CD_3_OD) and ^13^C NMR (150 MHz, CD_3_OD) data of Compounds **2** and **3**.

Position	2		3	
	*δ*_C_	*δ*_H_ (m, *J* in Hz)	*δ*_C_	*δ*_H_ (m, *J* in Hz)
2	163.4		163.4	
3	113.9	6.26 (d, 9.6)	114.0	6.26 (d, 9.3)
4	146.4	7.79 (d, 9.6)	146.4	7.80 (d, 9.3)
5	105.45	6.62, s	105.4	6.64, s
6	145.4		144.9	
7	138.5		138.3	
8	133.4		133.4	
9	138.6		138.7	
10	113.7		113.7	
1′	129.4		129.4	
2′	110.6	6.77 (d, 1.8)	105.4	6.79 (d, 1.8)
3′	145.9		150.5	
4′	135.2		135.2	
5′	150.4		145.9	
6′	105.52	6.80 (d, 1.8)	110.7	6.77 (d, 1.8)
7′	78.1	5.07 (d, 7.8)	78.0	5.08 (d, 7.8)
8′	79.9	4.21 (ddd, 7.8, 4.1, 2.6)	79.8	4.21 (ddd, 7.8, 3.8, 2.4)
9′	61.8	3.88 (dd, 12.6, 2.6) 3.60 (dd, 12.6, 4.1)	61.8	3.88 (dd, 12.3, 2.4) 3.60 (dd, 12.3, 3.8)
1″	128.5		128.5	
2″	104.1	6.59 (d, 1.8)	104.1	6.59 (d, 1.8)
3″	149.8		149.8	
4″	136.0		136.0	
5″	146.9		146.9	
6″	109.4	6.57 (d, 1.8)	109.4	6.57 (d, 1.8)
7″	77.7	4.84 (d, 7.8)	77.8	4.84 (d, 8.1)
8″	80.1	4.04 (ddd, 7.8, 4.4, 2.6)	80.1	4.03 (ddd, 8.1 4.5, 2.7)
9″	62.1	3.74 (dd, 12.6, 2.6) 3.54 (dd, 12.6, 4.4)	62.1	3.75 (dd, 12.6, 2.7) 3.54 (dd, 12.6, 4.5)
OCH_3_-5′	56.9	3.91, s	56.9	3.90, s
OCH_3_-3″	56.7	3.85, s	56.7	3.86, s

**Table 3:** ^1^H (600 MHz, CD_3_OD) and ^13^C NMR (150 MHz, CD_3_OD) data of Compound **4**.

Position	4	
	*δ*_C_	*δ*_H_ (m, *J* in Hz)
1α	55.7	2.56 (d, 13.5)
1β		3.11 (d, 13.5)
2	213.4	
3	83.0	4.07, s
4	45.8	
5	50.3	1.90 (dd, 12.0, 4.5)
6α	24.3	2.11 (ddd, 13.8, 4.5, 2.1)
6β		2.15 (ddd, 13.8, 12.0, 6.6)
7	129.0	5.83 (dt, 6.6, 2.1)
8	138.6	
9	55.6	2.40 (dt, 10.5, 2.1)
10	43.6	
11	68.1	3.82 (ddd, 12.0, 10.5, 5.8)
12a		2.01 (t, 12.0)
12b	40.1	1.53 (ddd, 12.0, 5.8, 2.1)
13	41.9	
14	81.1	3.59, s
15	147.2	5.94 (dd, 18.0, 11.1)
16a		5.02 (d, 18.0)
16b	112.4	5.01 (d, 11.1)
17	22.9	0.87, s
18	29.2	1.17, s
19	17.1	0.80, s
20	16.1	1.00, s

## Supplementary Materials

The following are available online, including 1D and 2D NMR spectra of new compounds **1**–**4**, the numerical value of bioactivity results, along with phytochemical data of known compounds **5**–**44**.
