# Supplementary material for: Chemical Constituents with GNMT-Promoter-Enhancing and NRF2-Reduction Activities from Taiwan Agarwood Excoecaria formosana
Source: Molecules. 2020 Apr 10;25(7):1746. doi: 10.3390/molecules25071746 (PMC7181199; doi:10.3390/molecules25071746)
Supplement: Supplementary file 1 [file molecules-25-01746-s001.pdf]

Chemical Constituents with GNMT-Promoter-Enhancing and NRF2-Reduction Activities from Taiwan Agarwood *Excoecaria formosana*

Ho-Cheng Wu [1], Ming-Jen Cheng [2], Chia-Hung Yen [1,3], Yi-Ming Arthur Chen [4,5], Yi-Siao Chen [6], Ih-Sheng Chen [7] and Hsun-Shuo Chang [1,3,7,8,*]

[1] Graduate Institute of Natural Products, College of Pharmacy, Kaohsiung Medical University, Kaohsiung 807, Taiwan; duncanwu762001@gmail.com (H.-C.W.); chyen@kmu.edu.tw (C.-H.Y.)

[2] Bioresource Collection and Research Center (BCRC), Food Industry Research and Development Institute (FIRDI), Hsinchu 300, Taiwan; cmj@firdi.org.tw (M.-J.C.)

[3] Drug Development and Value Creation Research Center, Kaohsiung Medical University, Kaohsiung 807, Taiwan

[4] Master Program in Clinical Pharmacogenomics and Pharmacoproteomics, College of Pharmacy, Taipei Medical University, Taipei 110, Taiwan; arthur@tmu.edu.tw (Y.-M.A.C.)

[5] Department of Medical Research and Education, Cheng Hsin General Hospital, Taipei 112, Taiwan

[6] Ph.D. Program in Environmental and Occupational Medicine, College of Medicine, Kaohsiung Medical University, Kaohsiung 807, Taiwan; dragonraja7992@yahoo.com.tw (Y.-S.C.)

[7] School of Pharmacy, College of Pharmacy, Kaohsiung Medical University, Kaohsiung 807, Taiwan; m635013@kmu.edu.tw (I.-S.C.)

[8] Department of Medical Research, Kaohsiung Medical University Hospital, Kaohsiung 807, Taiwan

* Correspondence: hschang@kmu.edu.tw (H.-S.C.); Tel.: +886-7-312-1101 (ext. 2664)

The List of Supplementary Material

Figure A1. ^1H NMR spectrum of (600 MHz, $CDCl_3$) spectrum of **1**......3
Figure A2. ^{13}C NMR spectrum of (150 MHz, $CDCl_3$) spectrum of **1**......3
Figure A3. DEPT spectrum of **1** 4
Figure A4. COSY spectrum of **1** 4
Figure A5. HMBC spectrum of **1** 5
Figure A6. ROESY spectrum of **1**......5
Figure A7. ^1H NMR spectrum of (600 MHz, CD_3OD) spectrum of **2** 6
Figure A8. ^{13}C NMR spectrum of (150 MHz, CD_3OD) spectrum of **2**......6
Figure A9. DEPT spectrum of **2** 7
Figure A10. COSY spectrum of **2** 7
Figure A11. HMBC spectrum of **2**......8
Figure A12. NOESY spectrum of **2** 8
Figure A13. ^1H NMR spectrum of (600 MHz, CD_3OD) spectrum of **3** 9
Figure A14. ^{13}C NMR spectrum of (150 MHz, CD_3OD) spectrum of **3**......9
Figure A15. DEPT spectrum of **3** 10
Figure A16. COSY spectrum of **3** 10
Figure A17. HMBC spectrum of **3**......11
Figure A18. NOESY spectrum of **3** 11
Figure A19. ^1H NMR spectrum of (600 MHz, CD_3OD) spectrum of **4** 12
Figure A20. ^{13}C NMR spectrum of (150 MHz, CD_3OD) spectrum of **4**......12
Figure A21. DEPT spectrum of **4** 13
Figure A22. COSY spectrum of **4** 13
Figure A23. HMBC spectrum of **4**......14
Figure A24. NOESY spectrum of **4** 14

Table A1. GNMT-promoter-enhancing activity (Fold of induction) of compounds from the whole plant of *E. formosana*......15
Table A2. NRF2 inhibition in Huh7 cells of compounds from the whole plant of *E. formosana* 16

Phytochemical data of known compounds **5–44**......17

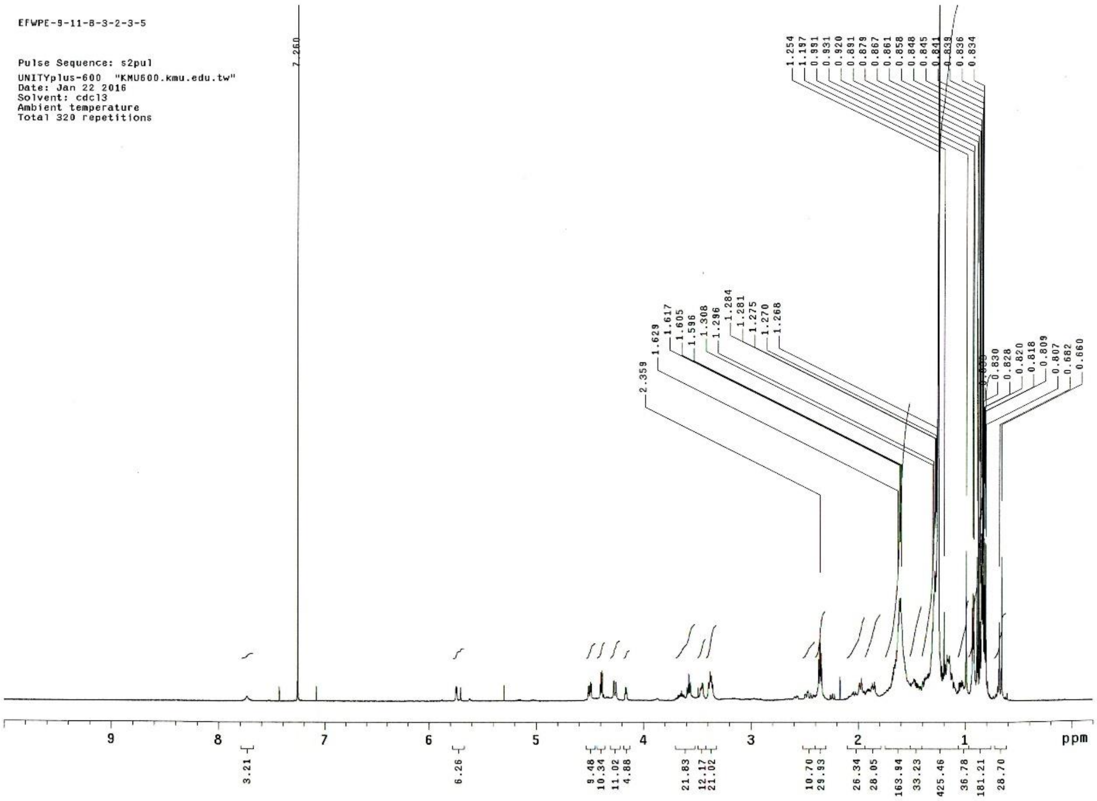

Figure A1. ^{1}H NMR spectrum of (600 MHz, $CDCl_3$) spectrum of **1**

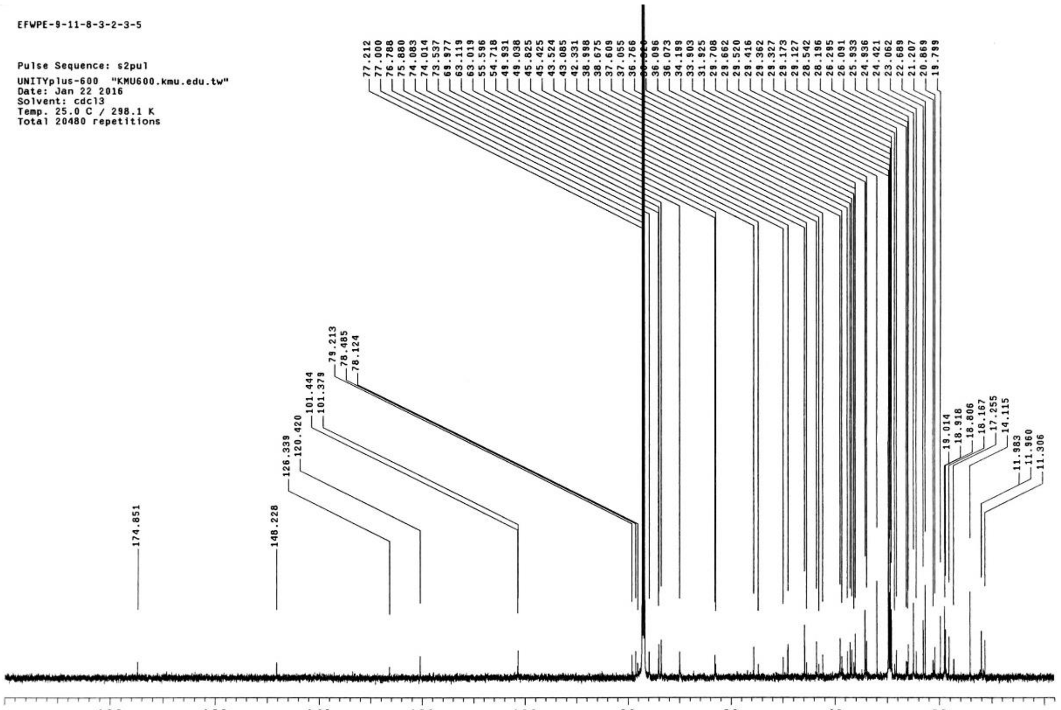

Figure A2. ^{13}C NMR spectrum of (150 MHz, $CDCl_3$) spectrum of **1**

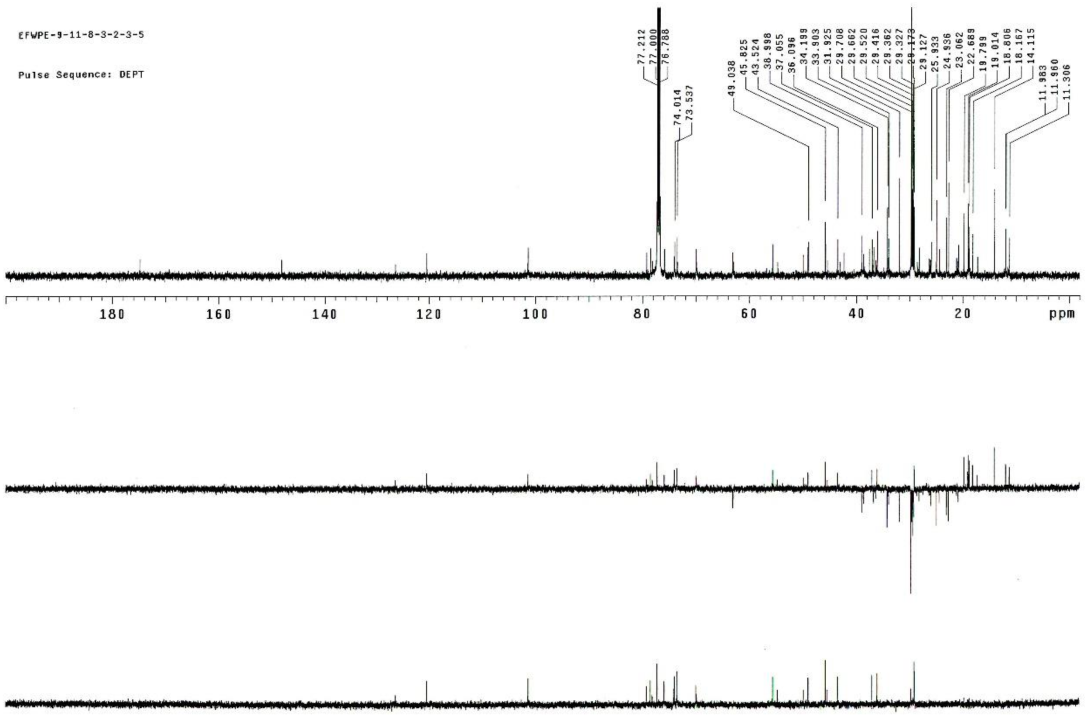

Figure A3. DEPT spectrum of **1**

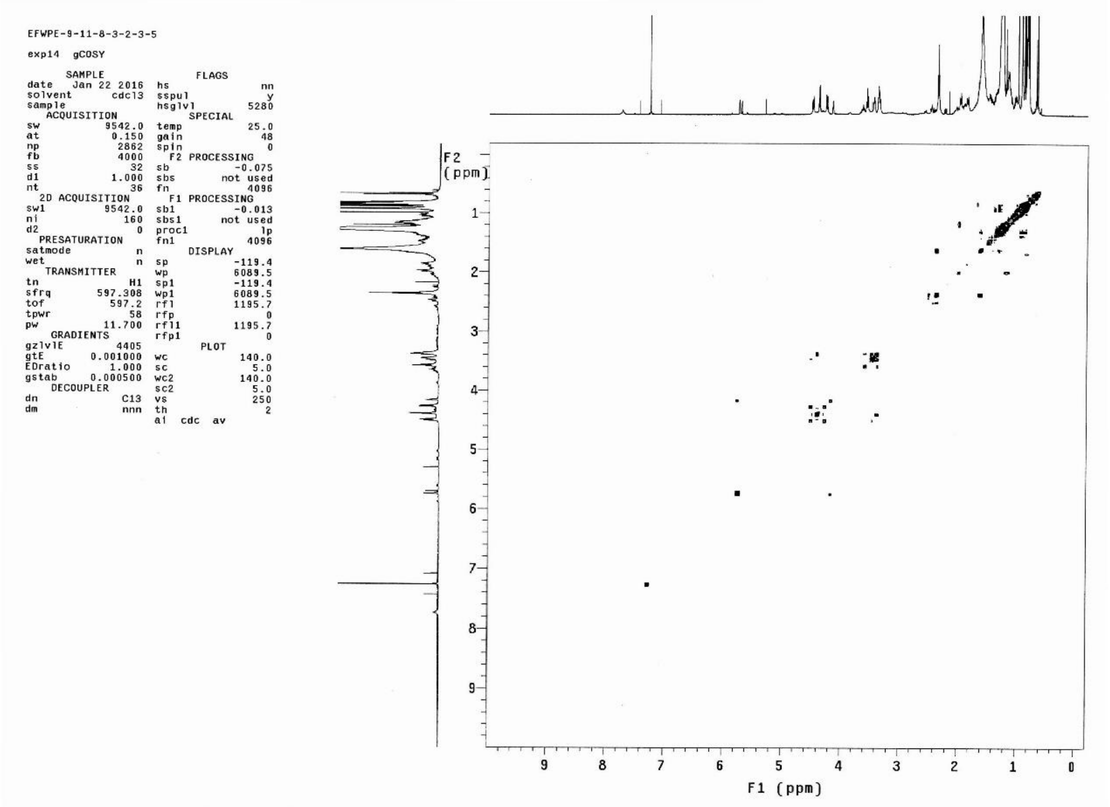

Figure A4. COSY spectrum of **1**

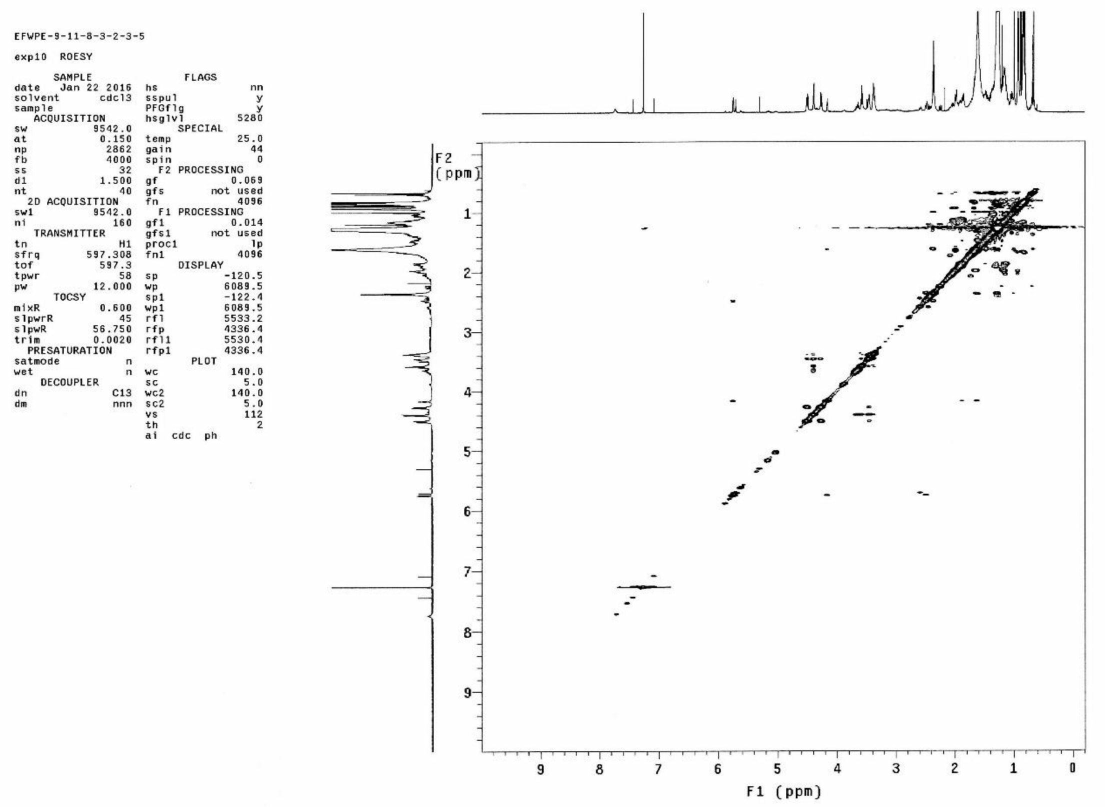

Figure A5. HMBC spectrum of **1**

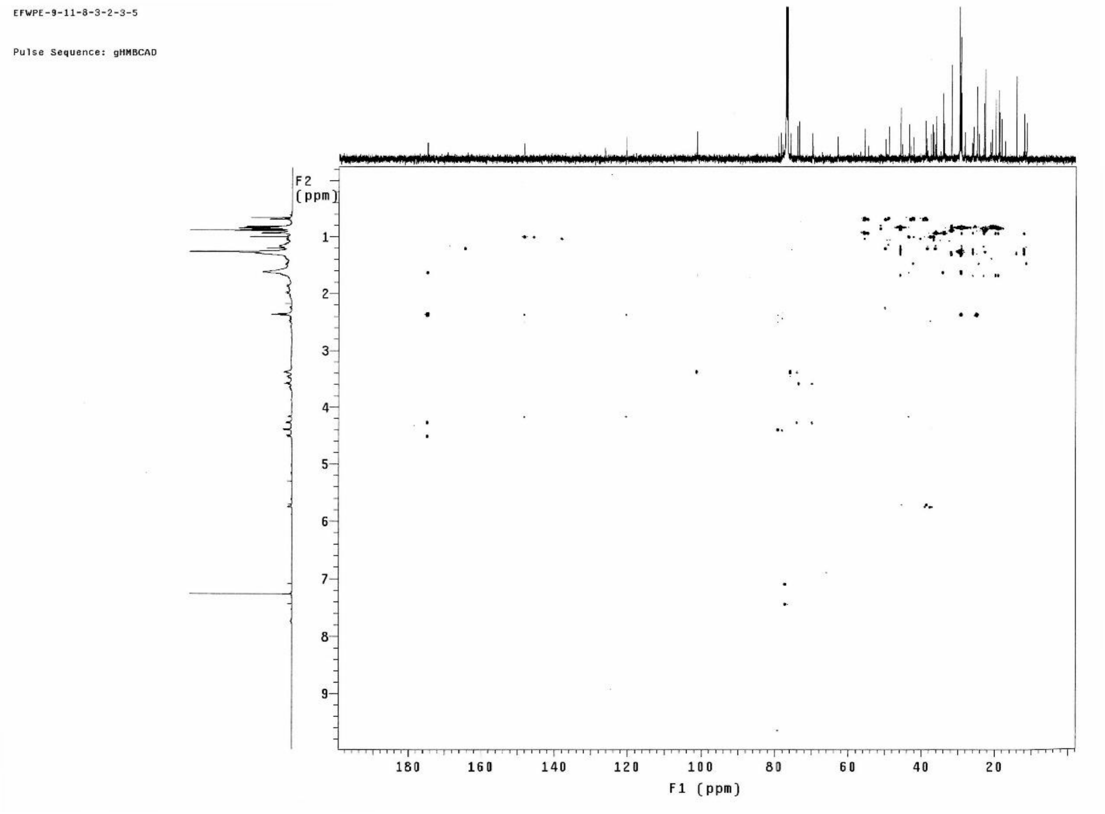

Figure A6. ROESY spectrum of **1**

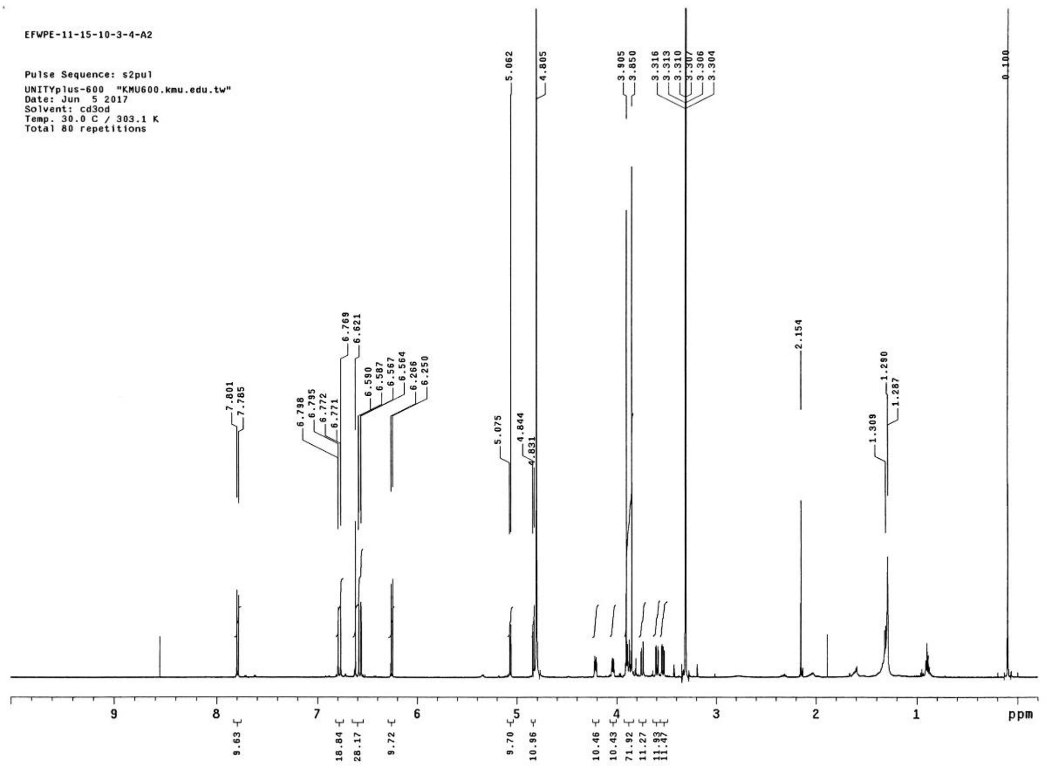

Figure A7. ^{1}H NMR spectrum of (600 MHz, CD_3OD) spectrum of **2**

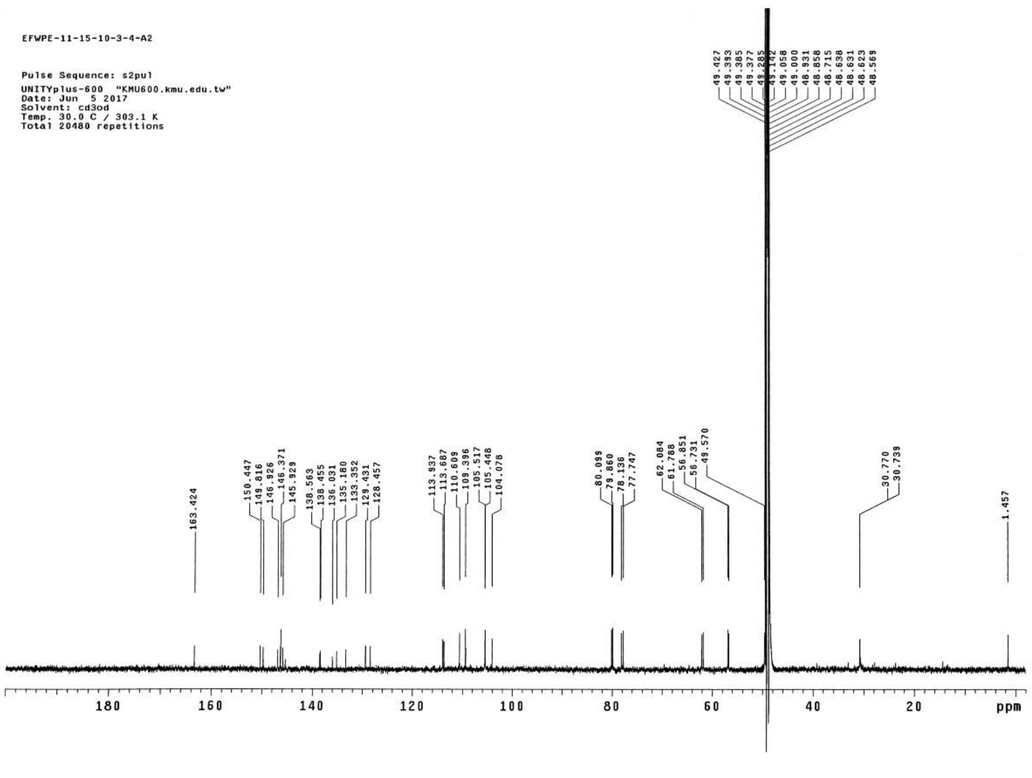

Figure A8. ^{13}C NMR spectrum of (150 MHz, CD_3OD) spectrum of **2**

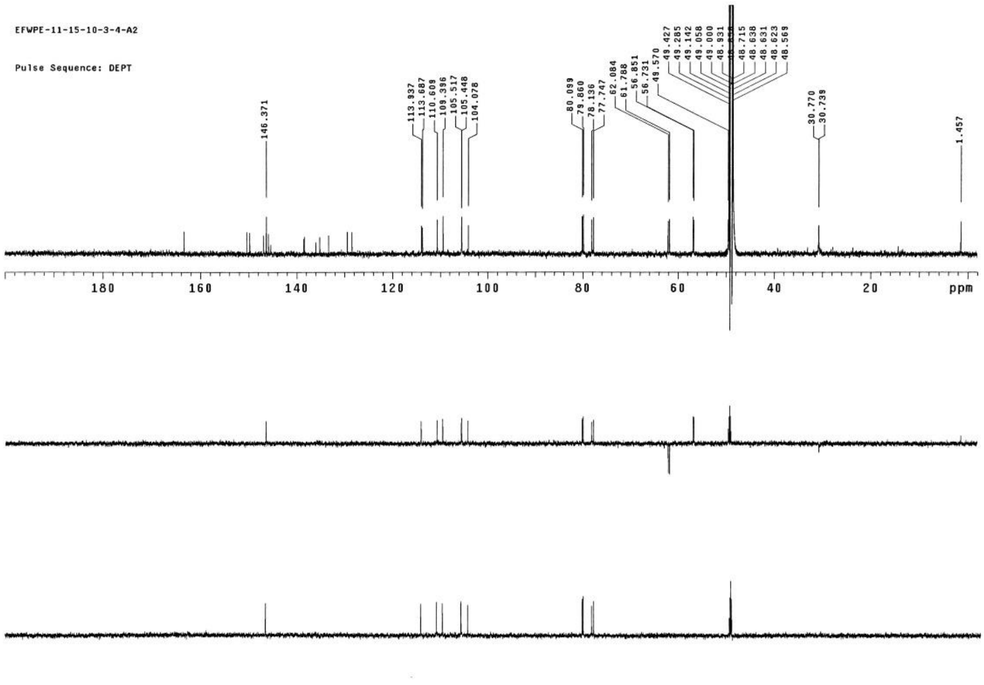

Figure A9. DEPT spectrum of **2**

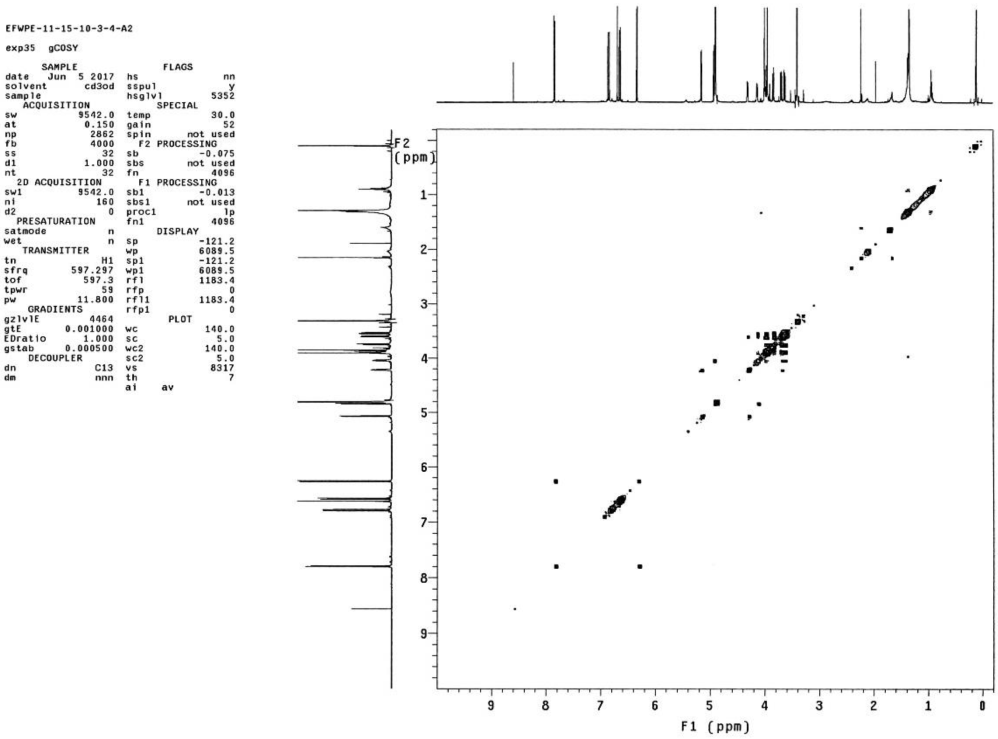

Figure A10. COSY spectrum of **2**

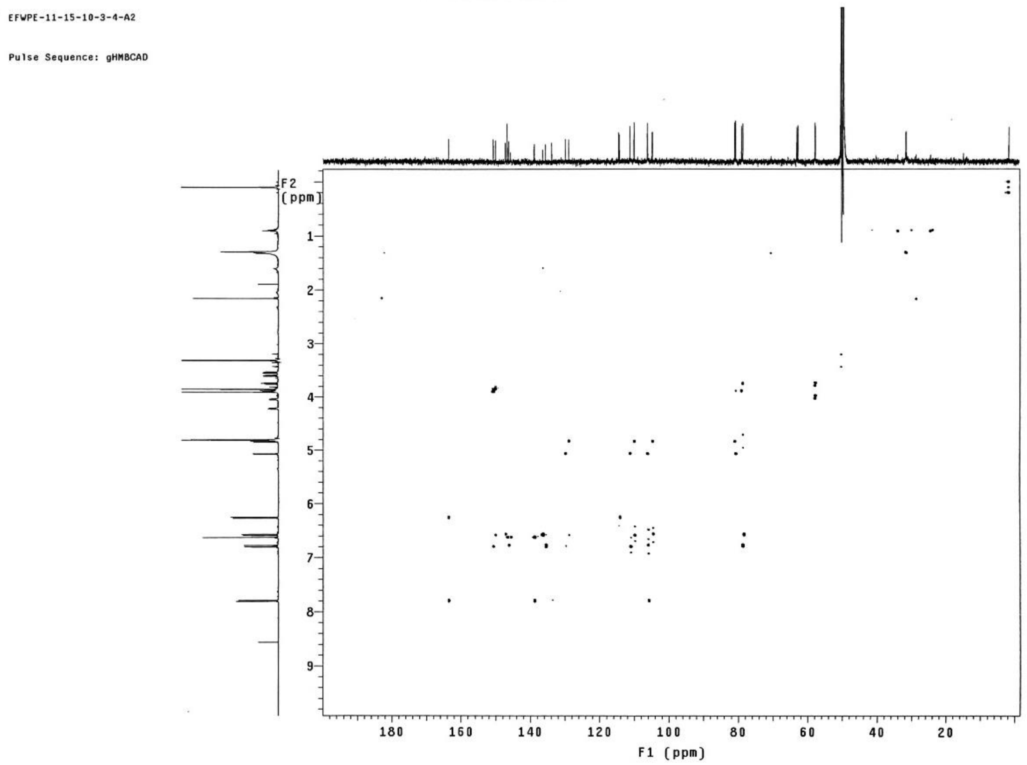

Figure A11. HMBC spectrum of **2**

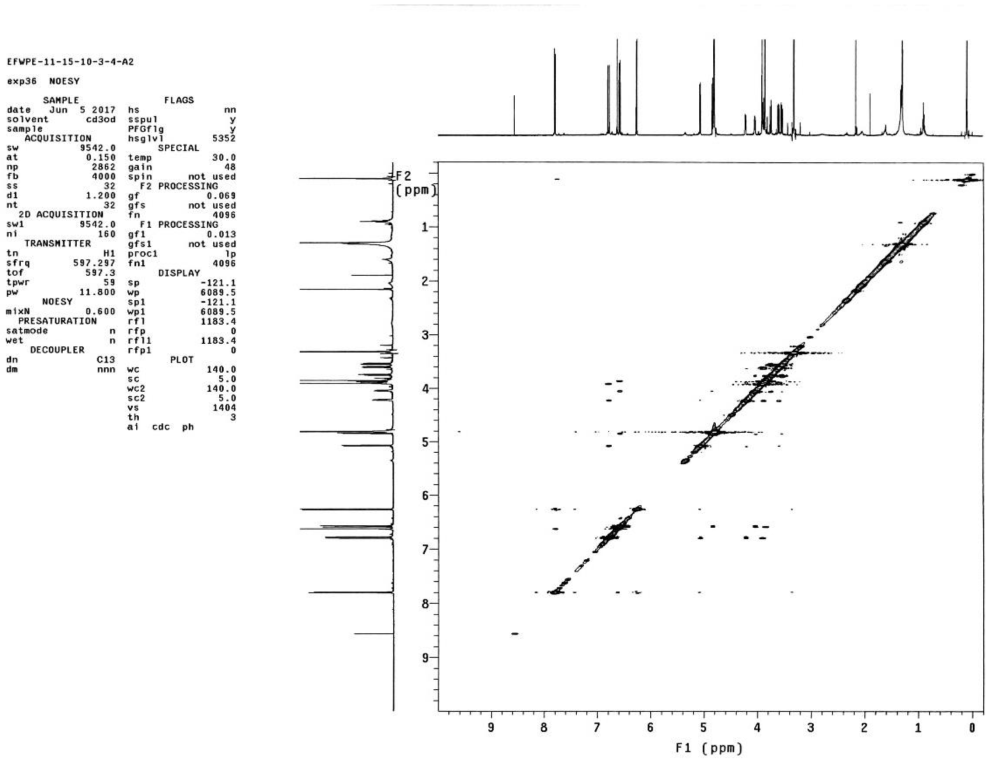

Figure A12. NOESY spectrum of **2**

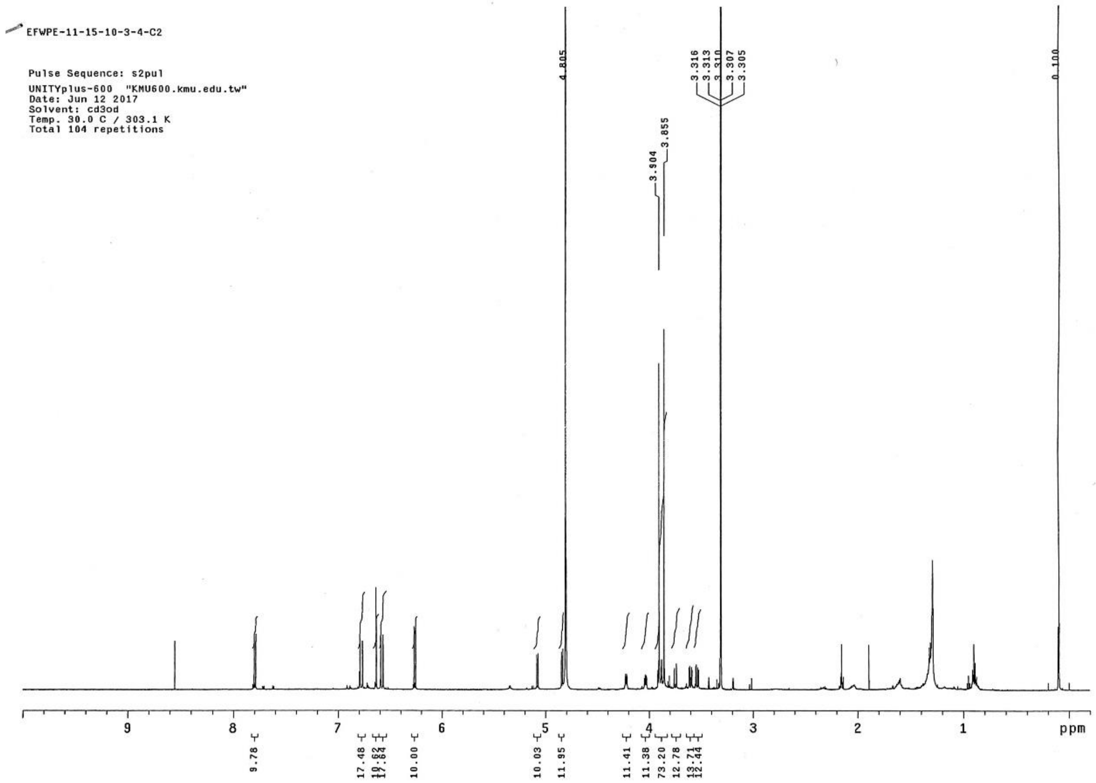

Figure A13. ^{1}H NMR spectrum of (600 MHz, CD_3OD) spectrum of **3**

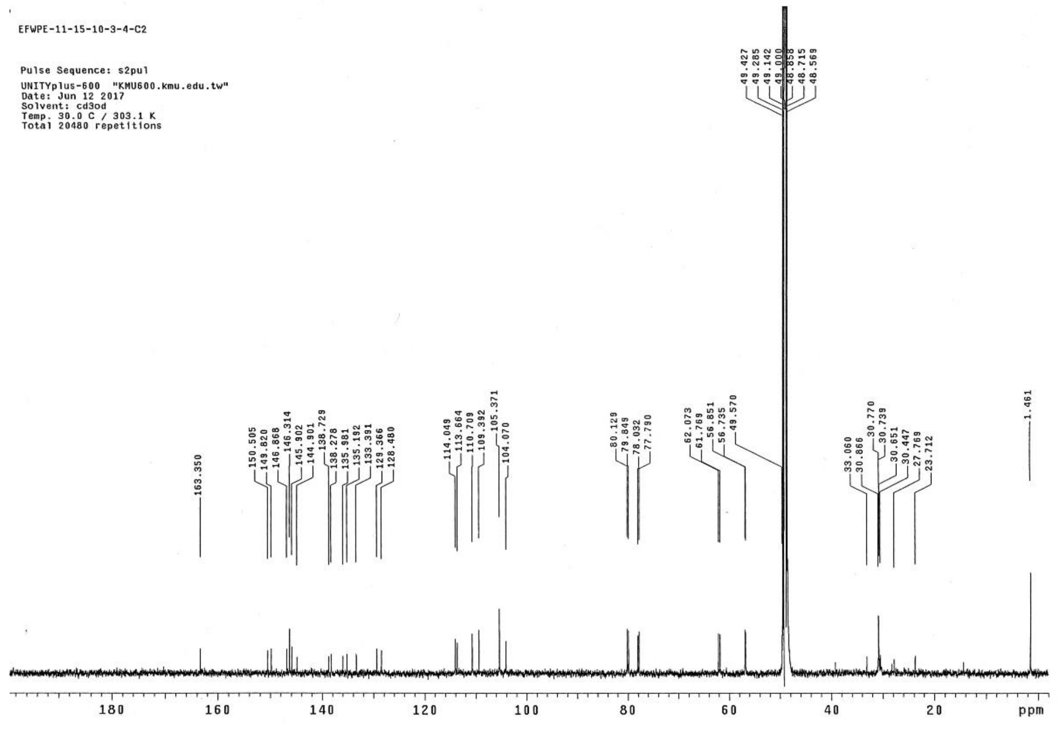

Figure A14. ^{13}C NMR spectrum of (150 MHz, CD_3OD) spectrum of **3**

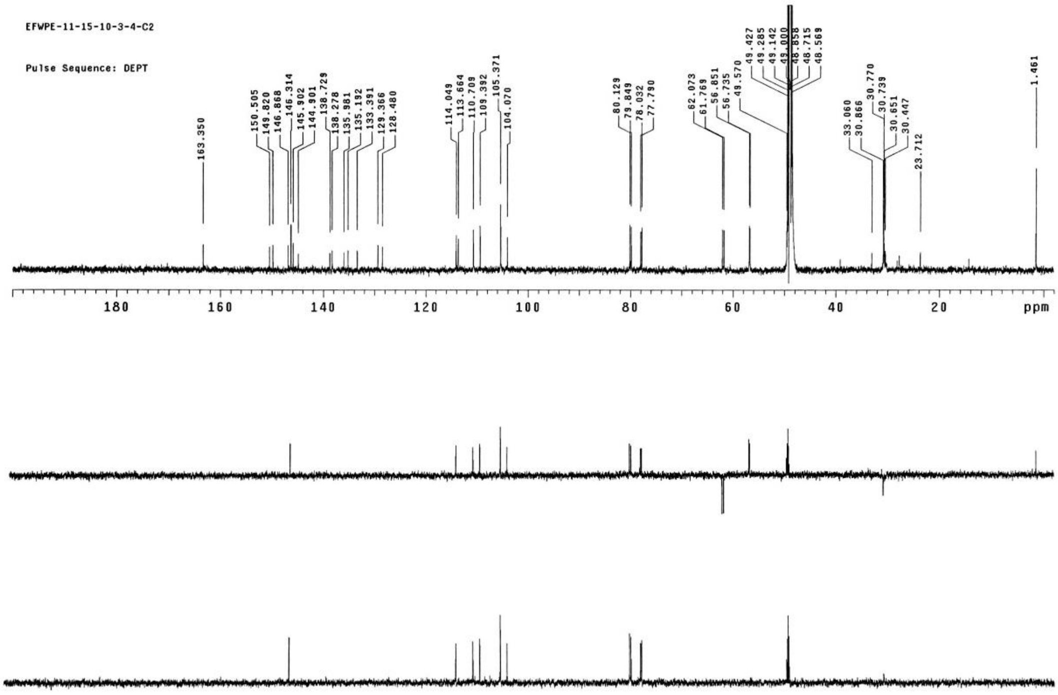

Figure A15. DEPT spectrum of **3**

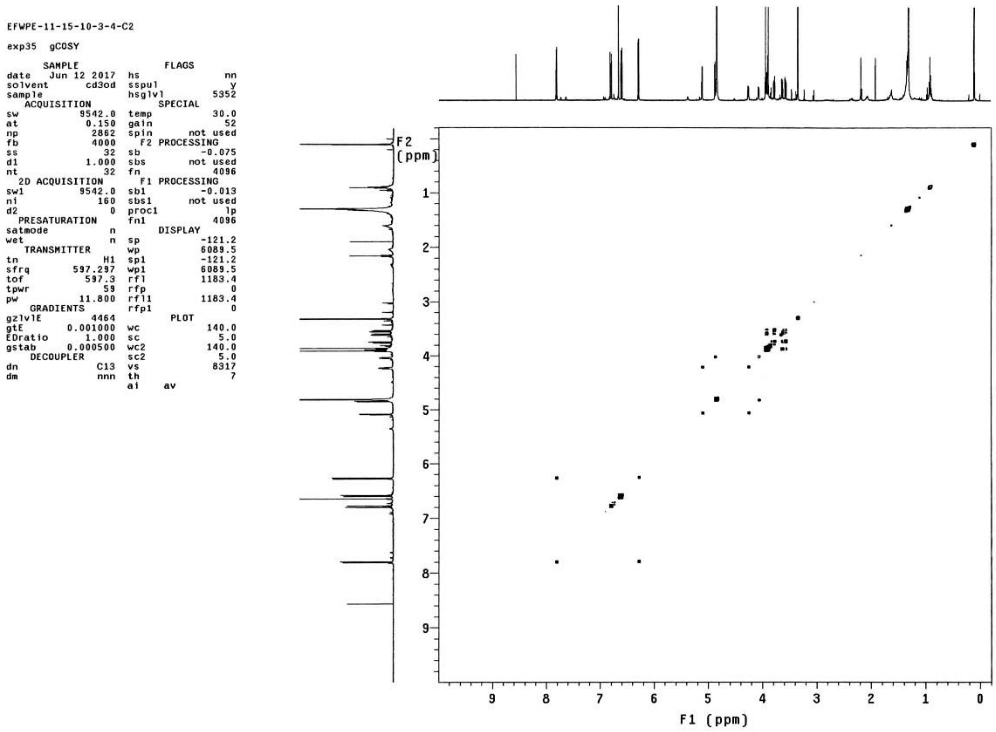

Figure A16. COSY spectrum of **3**

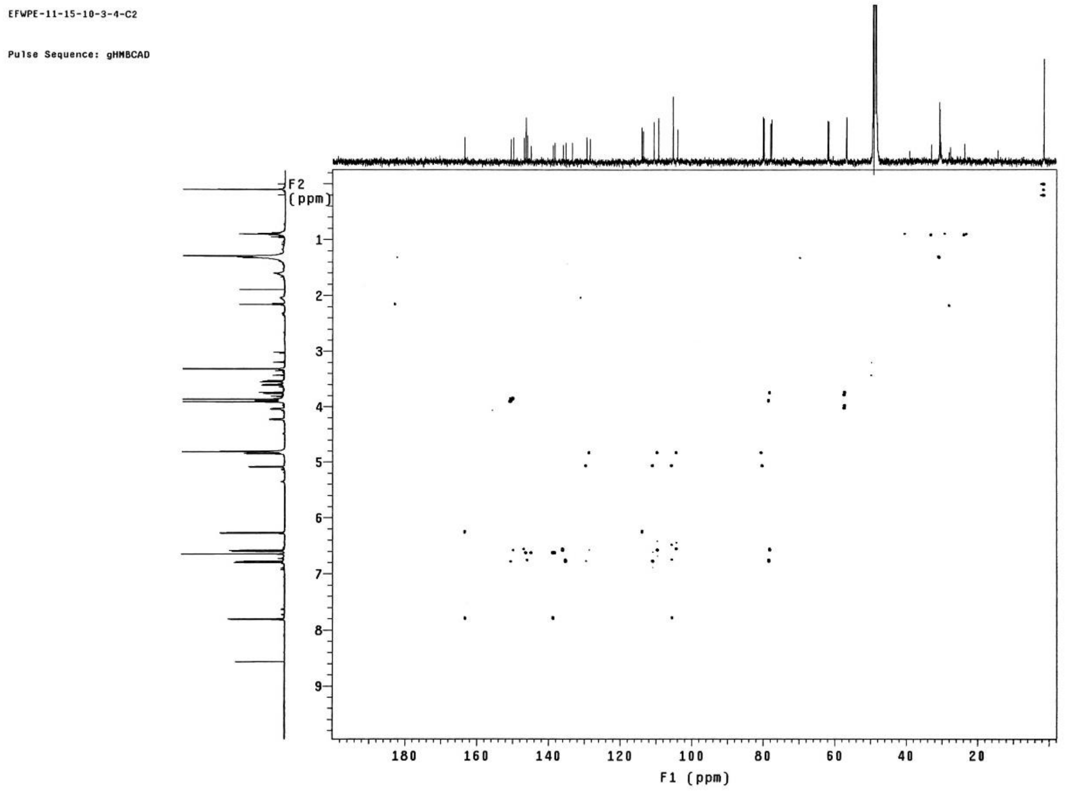

Figure A17. HMBC spectrum of **3**

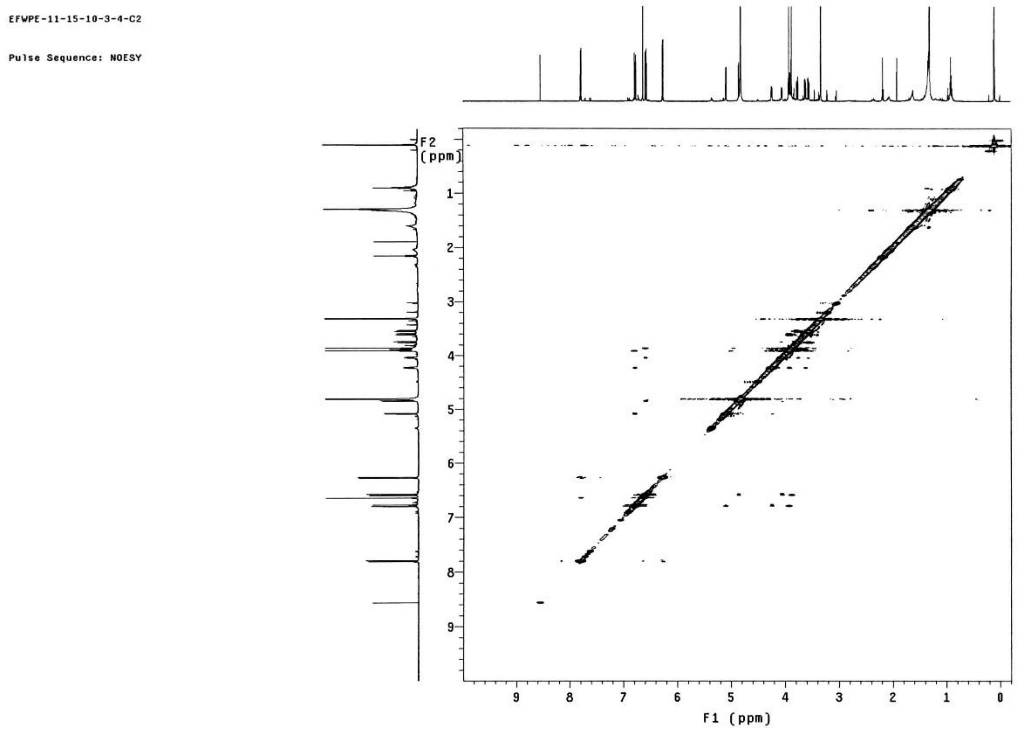

Figure A18. NOESY spectrum of **3**

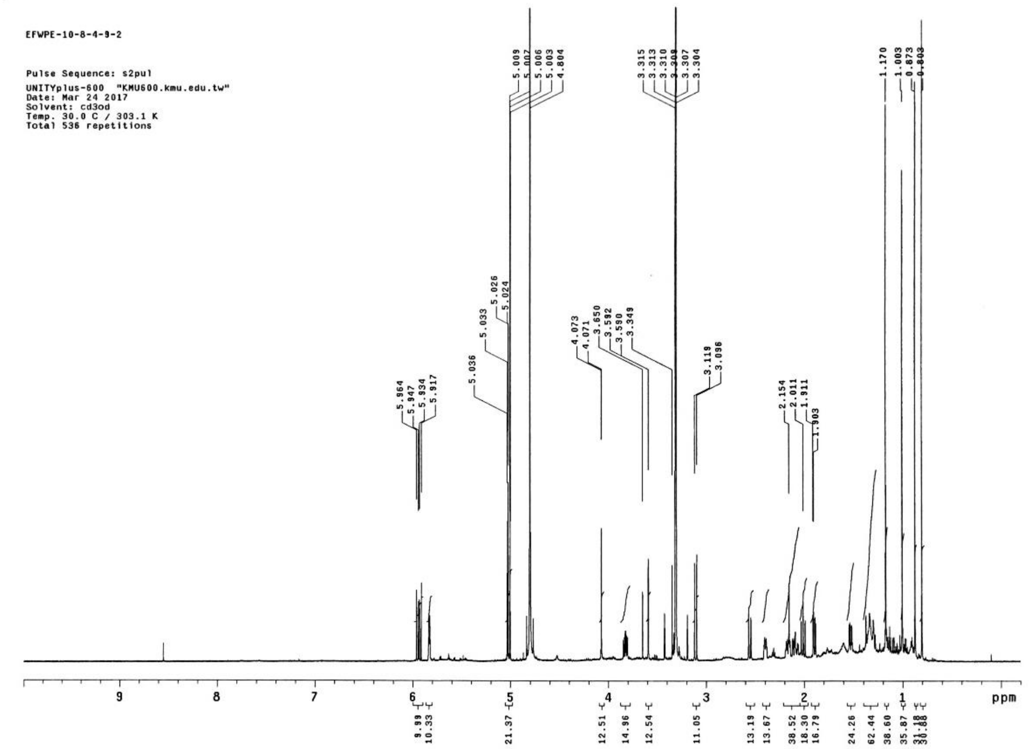

Figure A19. ^{1}H NMR spectrum of (600 MHz, CD_3OD) spectrum of **4**

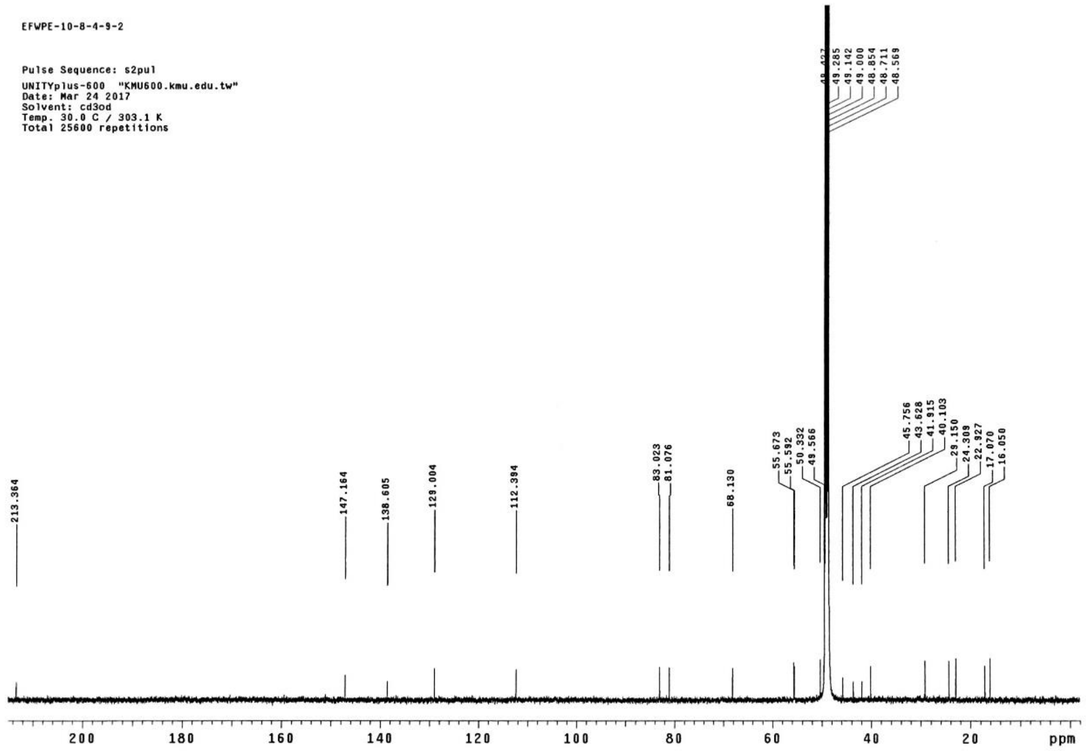

Figure A20. ^{13}C NMR spectrum of (150 MHz, CD_3OD) spectrum of **4**

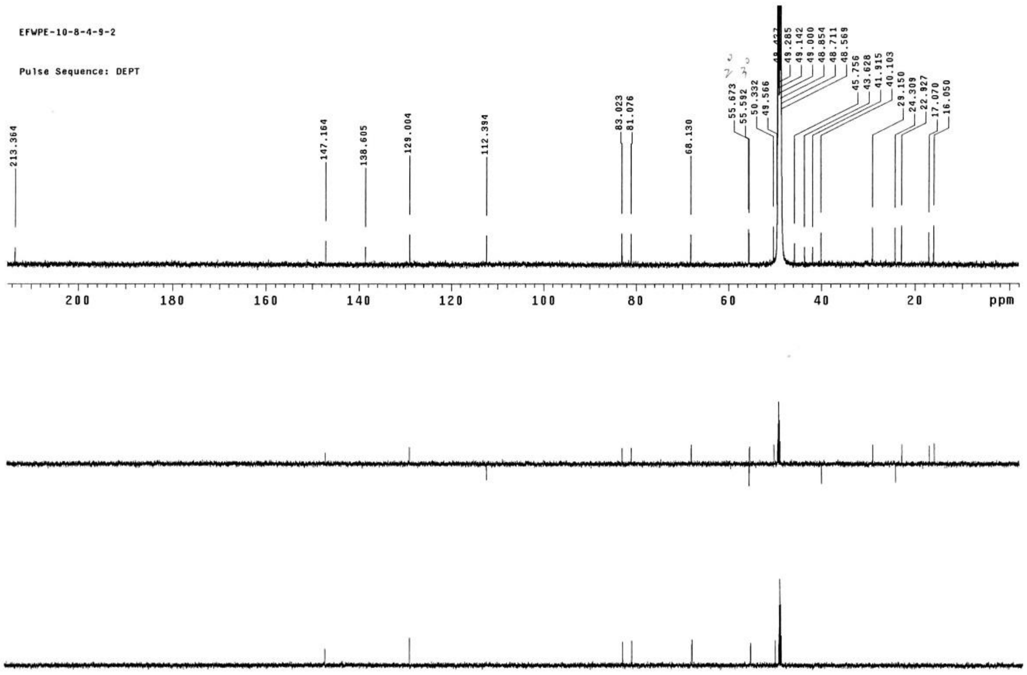

Figure A21. DEPT spectrum of **4**

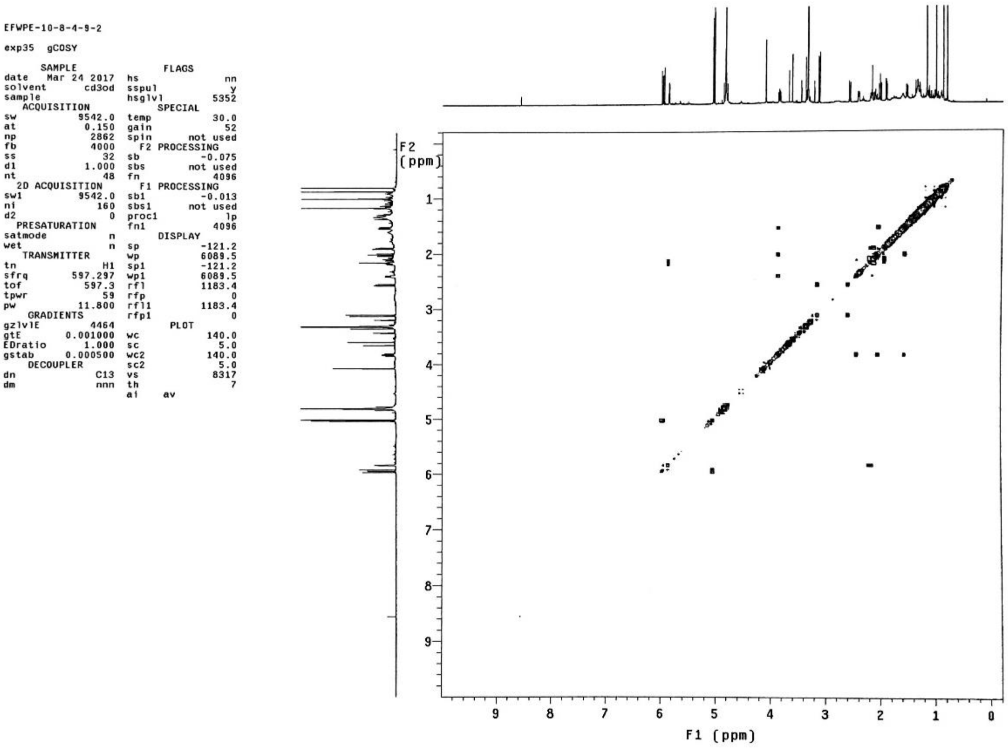

Figure A22. COSY spectrum of **4**

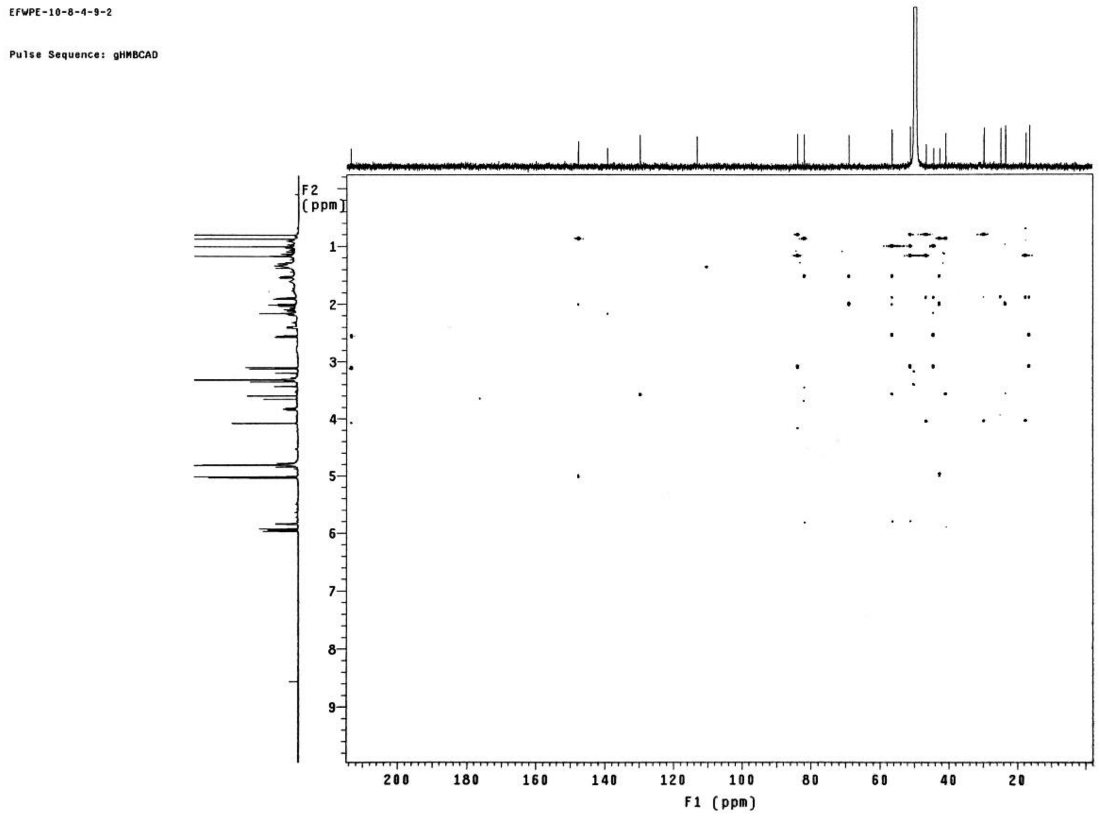

Figure A23. HMBC spectrum of **4**

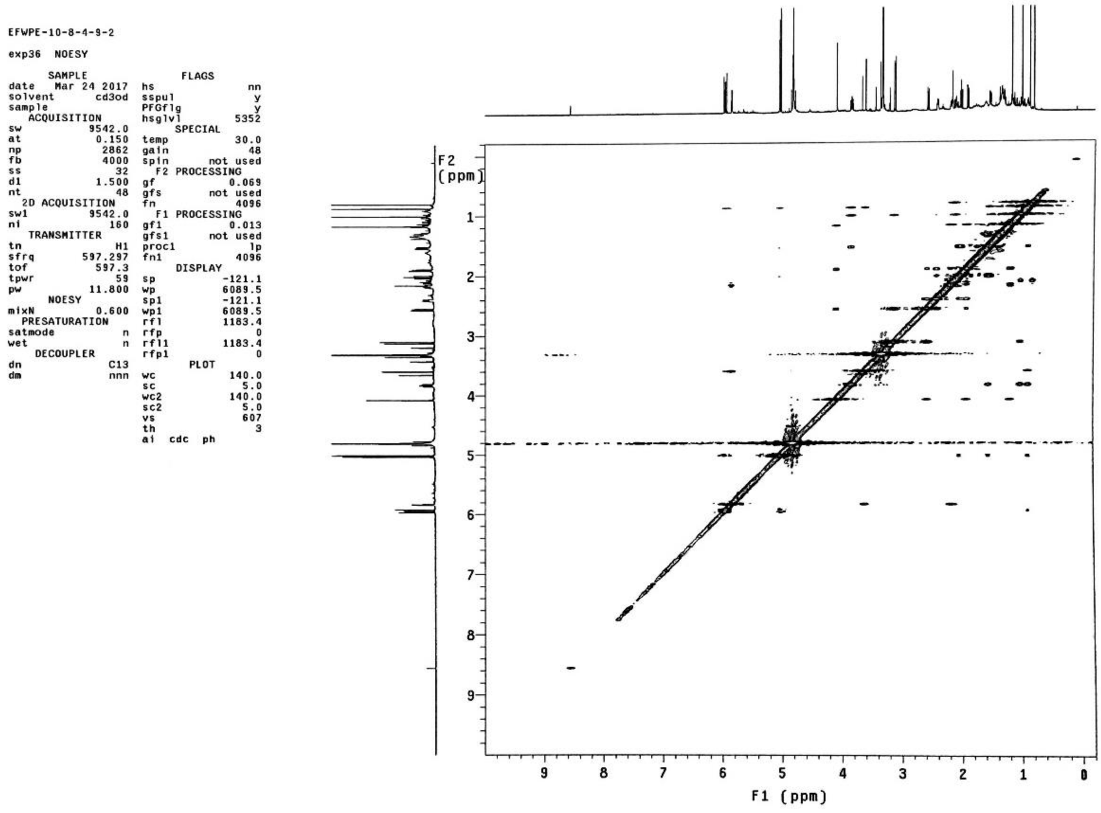

Figure A24. NOESY spectrum of **4**

Table A1. GNMT-promoter-enhancing activity (Fold of induction) of compounds from the whole plant of *E. formosana*

Compound[a]	Activity (Fold of induction)[b]	Compound[a]	Activity (Fold of induction)[b]
**1**	1.11±0.05	**26**	0.67±0.02
**2**	1.28±0.02	**27**	1.02±0.11
**3**	1.39±0.16	**28**	0.42±0.04
**4**	1.20±0.06	**29**	0.96±0.04
**5**	1.04±0.03	**31**	0.06±0.007
**6**	2.14±0.06	**32**	1.17±0.05
**7**	1.09±0.10	**33 & 34**	0.36±0.12
**9**	0.58±0.09	**35 & 36**	0.74±0.08
**10**	1.32±0.02	**37**	1.23±0.15
**12**	0.90±0.15	**38**	2.97±0.27
**13-16**	0.68±0.09	**39**	1.28±0.05
**17**	0.94±0.03	**40**	3.17±1.03
**18**	0.83±0.05	**41**	2.73±0.23
**19**	1.29±0.05	**42**	2.63±0.14
**20**	1.46±0.07	**43**	6.57±0.13
**22**	0.68±0.07	**44**	2.62±0.05
**24**	0.91±.0.10	PGG[c]	4.45±0.26
**25**	0.81±0.04		

[a]Sample concentration is 100 μM. [b]GNMT promoter activity (Fold of induction) = observed activity/ solvent control activity. [c]PGG = 1,2,3,4,6-penta-*O*-galloyl-β-D-glucose was used as a positive control for GNMT activation with 100 μM.

Table A2. NRF2 inhibition in Huh7 cells of compounds from the whole plant of *E. formosana*

Compound[a]	Activity (Fold of induction)[b]	Compound[a]	Activity (Fold of induction)[b]
**1**	60.5±1.5	**26**	69.9±0.3
**2**	81.9±8.1	**27**	101.8±1.5
**3**	89.7±2.1	**28**	83.0±2.3
**4**	93.5±3.9	**29**	104.5±9.2
**5**	89.2±4.5	**31**	88.8±3.5
**6**	91.8±1.1	**32**	84.7±4.0
**7**	79.4±5.1	**33 & 34**	93.3±3.7
**9**	93.2±0.7	**35 & 36**	99.2±1.4
**10**	85.6±5.9	**37**	109.7±1.7
**12**	80.4±1.9	**38**	75.7±0.9
**13-16**	NT[c]	**39**	95.7±2.5
**17**	89.8±1.5	**40**	33.1±0.2
**18**	116.2±2.7	**41**	67.8±3.9
**19**	91.5±5.1	**42**	59.2±2.9
**20**	91.3±2.4	**43**	45.2±2.5
**22**	103.7±2.8	**44**	73.0±2.5
**24**	104.9±2.6	Retinoic acid[d]	34.2±1.4
**25**	110.0±5.3		

[a]Sample concentration is 100 μM. [b]Relative NRF2 activity was presented as the percentage to solvent control. [c]NT, None tested. [d]Retinoic acid was used as a positive control for Nrf2 inhibition with 1μM.

Phytochemical data of known compounds 5–44

Deglucosyl lauroside B (5): Colorless oil; $[\alpha]^{26}_{D}$: –15 (c 0.16, MeOH); IR υ_{max} (ATR) 3381 (OH), 1698 (C=O) cm^{-1}; ^{1}H NMR (CD_3OD, 600 MHz) *δ*: 0.909 (3H, s, H-12), 0.914 (3H s, H-11), 1.28 (3H, d, *J* = 6.3 Hz, H-10), 1.83 (1H, dd, *J* = 13.8, 2.1 Hz, H-2b), 2.18 (1H, dddd, *J* = 14.1, 4.8, 4.8, 3.0 Hz, H-5), 2.26 (1H, ddd, *J* = 14.1, 4.8, 2.1 Hz, H-4b), 2.83 (1H, t, *J* = 14.1 Hz, H-4a), 2.93 (1H, d, *J* = 13.8 Hz, H-2a), 3.59 (1H, dd, *J* = 11.4, 3.0 Hz, H-13b), 3.84 (1H, dd, *J* = 11.4, 4.8 Hz, H-13a), 4.36 (1H, quint, *J* = 6.3, 1.2 Hz, H-9), 5.75 (1H, dd, *J* = 15.6, 1.2 Hz, H-7), 5.95 (1H, dd, *J* = 15.6, 6.3 Hz, H-8); ESI-MS *m*/*z*: 265 [M+Na]$^+$

Gallic acid (6): Colorless needles (CH_2Cl_2-MeOH); IR υ_{max} (ATR) 3107 (OH), 1684 (C=O), 1612, 1539 (aromatic ring) cm^{-1}; ^{1}H NMR (acetone-d_6, 400 MHz) *δ*: 7.15 (2H, s, H-2 and H-6); ^{13}C NMR (acetone-d_6, 100 MHz) ***δ*:** 110.2 (C-2 and C-6), 122.2 (C-1), 138.7 (C-4), 146.1 (C-3 and C-5), 167.7 (C-7); ESI-MS *m*/*z*: 193 [M+Na]$^+$

Methyl gallate (7): Colorless needles (CH_2Cl_2-MeOH); IR υ_{max} (ATR) 3464 (OH), 1686 (C=O), 1617, 1542, 1438 (aromatic ring) cm^{-1}; ^{1}H NMR (acetone-d_6, 400 MHz) *δ*: 3.78 (3H, s, OCH_3), 7.11 (2H, s, H-2 and H-6); ^{13}C NMR (acetone-d_6, 100 MHz) *δ*: 51.1 (OCH_3), 108.9 (C-2 and C-6), 120.9 (C-1), 137.9 (C-4), 145.2 (C-3 and C-5), 166.4 (C=O); ESI-MS *m*/*z*: 185 [M+H]$^+$

4-Methoxybenzoic acid (8): Colorless oil; UV (MeOH) λ_{max} (log *ε*) 210 (3.72), 255 (3.57) nm; IR υ_{max} (ATR) 3436 (OH), 1710 (C=O), 1607, 1441 (aromatic ring) cm^{-1}; ^{1}H NMR ($CDCl_3$, 400 MHz) *δ*: 3.88 (3H, s, OCH_3-4), 6.85 (2H, d, *J* = 8.8 Hz, H-3 and H-5), 7.96 (2H, d, *J* = 8.8 Hz, H-2 and H-6); ESI-MS *m*/*z*: 153 [M+H]$^+$

3-Hydroxy-1-(3,5-dimethoxy-4-hydroxyphenyl)propan-1-one (9): Whitish powder; UV (MeOH) λ_{max} (log ε) 216 (3.55), 234 (3.41), 298 (3.30) nm; IR υ_{max} (ATR) 3370 (OH), 1659 (C=O), 1591, 1515, 1453 (aromatic ring) cm^{-1}; ^{1}H NMR (CD_3OD, 400 MHz) *δ*: 3.17 (2H, t, *J* = 4.2 Hz, H-8), 3.90 (6H, s, OCH_3-2 and OCH_3-6), 3.95 (2H, t, *J* = 4.2 Hz, H-9), 7.32 (2H, s, H-3 and H-5); ^{13}C NMR (CD_3OD, 100 MHz) *δ*: 41.7 (C-8), 56.9 (OCH_3-2 and OCH_3-6), 59.1 (C-9), 107.6 (C-3 and C-5), 129.2 (C-1), 144.9 (C-4), 149.5 (C-2 and C-6), 200.0 (C-7); ESI-MS *m*/*z*: 227.17 [M+H]$^+$

3-Hydroxy-1-(4-hydroxy-3-methoxyphenyl)propan-1-one (10): Yellowish solid; UV (MeOH) λ_{max} (log *ε*) 205 (4.11), 228 (3.77), 275 (3.66) nm; IR υ_{max} (ATR) 3310 (OH), 1659 (C=O), 1591, 1516, 1453 (aromatic ring) cm^{-1}; IR υ_{max} (ATR) 3310 (OH), 1714 (C=O) cm^{-1}; ^{1}H NMR (CD_3OD, 600 MHz) *δ*: 3.16 (2H, t, *J* = 6.2 Hz, H-8), 3.90 (3H, s, OCH_3-2), 3.94 (2H, t, *J* = 6.2 Hz, H-9), 6.87 (1H, d, *J* = 8.0 Hz, H-6), 7.55 (1H, d, *J* = 2.0 Hz, H-3), 7.58 (1H, dd, *J* = 8.0, 2.0 Hz, H-5); ^{13}C NMR (CD_3OD, 150 MHz) *δ*: 41.7 (C-8), 56.4 (OCH_3-2), 59.0 (C-9), 112.0 (C-3), 115.8 (C-6), 124.7 (C-5), 130.7 (C-4), 149.1 (C-2), 153.4 (C-1), 199.7 (C-7); ESI-MS *m*/*z*: 197 [M+H]$^+$

2,3-Dihydroxy-1-(4-hydroxy-3-methoxyphenyl)propan-1-one (11): Whitish solid; UV (MeOH) λ_{max} (log *ε*) 205 (3.66), 280 (3.25) nm; IR υ_{max} (ATR) 3327 (OH), 1664 (C=O), 1590, 1517, 1424 (aromatic ring) cm^{-1}; ^{1}H NMR (CD_3OD, 400 MHz) *δ*: 3.73 (1H, dd, *J* = 11.7, 5.2 Hz, H-9b), 3.89 (1H, dd, *J* = 11.7, 3.9 Hz, H-9a), 3.92 (3H, s, OCH_3-2), 5.11 (1H, dd, *J* = 5.2, 3.9 Hz, H-8), 6.88 (1H, d, *J* = 8.4 Hz, H-6), 7.58 (1H, d, *J* = 2.0 Hz, H-3), 7.59 (1H, dd, *J* = 8.4, 2.0 Hz, H-5); ESI-MS *m*/*z*: 213 [M+H]$^+$

(2*S*,3*R*)-4*E*-Dehydrochebulic acid trimethyl ester (12): Colorless needles; $[\alpha]^{26}_{D}$: –34 (*c* 0.205, MeOH); UV (MeOH) λ_{max} (log *ε*) 220 (4.34), 285 (3.80) nm; IR υ_{max} (ATR) 3400 (OH), 1712 (C=O), 1608, 1492, 1438 (aromatic ring) cm^{-1}; ^{1}H NMR (acetone-d_6, 400 MHz) *δ*: 3.62 (3H, s, OCH_3-6 or OCH_3-7), 3.63 (3H, s, OCH_3-7 or OCH_3-6), 3.67 (3H, s, OCH_3-1), 5.27 (1H, d, *J* = 1.4 Hz, H-2), 5.40 (1H, d, *J* = 1.4 Hz, H-3), 6.81 (1H, s, H-5), 7.13 (1H, s, H-3'); ^{13}C NMR (acetone-d_6,

100 MHz) δ: 36.0 (C-3), 53.0 (OCH_3-6 or OCH_3-7), 53.4 (OCH_3-7 or OCH_3-6), 53.8 (OCH_3-1), 79.8 (C-2), 109.3 (C-3'), 116.7 (C-1'), 119.2 (C-2'), 130.4 (C-5), 139.6 (C-4'), 143.5 (C-4), 144.3 (C-6'), 146.7 (C-5'), 164.3 (C-7'), 166.7 (C-6), 167.6 (C-7), 171.1 (C-1); ESI-MS *m/z*: 397 $[M+H]^+$

A mixture of gynuramides I~IV (13~16): Whitish solid; $[\alpha]^{26}_D$: +11 (*c* 0.75, pyridine); IR υ_{max} (ATR) 3338 (OH), 1633 (amide) cm^{-1}; 1H NMR (pyridine-d_5, 400 MHz) δ: 0.87 (6H, t, *J* = 7.4 Hz, terminal methyl), 1.76 (2H, m, H-11), 2.00 (2H, m, H-7), 2.07 (1H, m, H-3'b), 2.13 (2H, m, H-5), 2.16 (2H, m, H-10), 2.31 (1H, m, H-3'a), 4.29 (1H, m, H-4), 4.36 (1H, m, H-3), 4.43 (1H, dd, *J* = 10.8, 4.8 Hz, H-1b), 4.52 (1H, dd, *J* = 10.8, 4.8 Hz, H-1a), 4.63 (1H, dd, *J* = 7.8, 3.8 Hz, H-2'), 5.13 (1H, quint, *J* = 4.8 Hz, H-2), 5.52 (2H, m, H-8 and H-9), 8.60 (1H, d, *J* = 9.2 Hz, NH); 13C NMR (pyridine-d_5, 100 MHz) δ: 14.3 (terminal methyl), 22.9 (C-17), 25.8 (C-11), 26.7 (C-4' and C-6), 29.5~30.2 (all CH_2), 32.1 (C-16), 32.9 (C-7), 33.3 (C-10), 33.8 (C-5), 35.7 (C-3'), 52.9 (C-2), 61.9 (C-1), 72.4 (C-2'), 72.9 (C-4), 76.8 (C-3), 130.7 (C-9), 130.8 (C-8), 175.3 (C-1'); gynuramide I (**13**): 718 $[M+Na]^+$, gynuramide II (**14**): 704 $[M+Na]^+$, gynuramide III (**15**): 690 $[M+Na]^+$, gynuramide IV (**16**): 676 $[M+Na]^+$

Scopoletin (17): Yellowish powder; UV (MeOH) λ_{max} (log ε) 209 (4.19), 228 (4.04), 262 (3.53), 297 (3.61), 344 (3.94) nm; IR υ_{max} (ATR) 3335 (OH), 1702 (C=O), 1607, 1564, 1511 (aromatic ring) cm^{-1}; ^{1}H NMR (acetone-d_6, 400 MHz) δ: 3.89 (3H, s, OCH_3-6), 6.14 (1H, d, *J* = 9.4 Hz, H-3), 6.78 (1H, s, H-8), 7.17 (1H, s, H-5), 7.83 (1H, d, *J* = 9.4 Hz, H-4); ESI-MS *m/z*: 193 $[M+H]^+$

Fraxetin (18): Yellowish solid; UV (MeOH) λ_{max} (log ε) 210 (4.4), 340 (3.86) nm; IR υ_{max} (ATR) 3360 (OH), 1680 (C=O), 1575, 1508, 1456 (aromatic ring) cm^{-1}; ^{1}H NMR (CD_3OD, 400 MHz) δ: 3.90 (3H, s, OCH_3-7), 6.21 (1H, d, *J* = 9.6 Hz, H-3), 6.72 (1H, s, H-8), 7.84 (1H, d, *J* = 9.6 Hz, H-4); ESI-MS *m/z*: 209 $[M+H]^+$

6-Hydroxy-5,7-dimethoxycoumarin (19): Yellowish powder; IR υ_{max} (ATR) 3402 (OH), 1711 (C=O), 1580, 1459, 1415 (aromatic ring) cm^{-1}; ^{1}H NMR (CD_3OD, 400 MHz) δ: 3.85 (3H, s, H-7), 3.90 (3H, s, H-5), 6.00 (1H, d, *J* = 9.2 Hz, H-3), 6.77 (1H, s, H-8), 7.79 (1H, d, *J* = 9.2 Hz, H-4); ESI-MS *m/z*: 223 $[M+H]^+$

Cleomiscosin A (20): Whitish solid; UV (MeOH) λ_{max} (log ε) 205 (4.69), 325 (4.02) nm; IR υ_{max} (ATR) 3308 (OH), 1696 (C=O), 1612, 1571, 1523, 1447 (aromatic ring) cm^{-1}; ^{1}H NMR (pyridine-d_5, 400 MHz) δ: 3.71 (3H, s, OCH_3-3'), 3.80 (3H, s, OCH_3-6), 3.91 (1H, ddd, *J* = 13.0, 6.0, 2.4 Hz, H-9'b), 4.32 (1H, ddd, *J* = 13.0, 6.0, 2.4 Hz, H-9'a), 4.48 (1H, dt, *J* = 8.2, 2.4 Hz, H-8'), 5.59 (1H, d, *J* = 8.2 Hz, H-7'), 6.44 (1H, d, *J* = 9.4 Hz, H-3), 6.73 (1H, s, H-5), 7.30 (1H, d, *J* = 8.0 Hz, H-5'), 7.36 (1H, dd, *J* = 8.2, 2.0 Hz, H-6'), 7.42 (1H, d, *J* = 2.0 Hz, H-2'), 7.55 (1H, t, *J* = 6.0 Hz, OH-9', D_2O exchangable), 7.75 (1H, d, *J* = 9.4 Hz, H-4), 11.19 (1H, s, OH-4', D_2O exchangable); ESI-MS *m/z* 409 $[M+Na]^+$

Cleomiscosin B (21): Whitish solid; UV (MeOH) λ_{max} (log ε) 205 (4.69), 325 (4.02) nm; IR υ_{max} (ATR) 3424 (OH), 1708 (C=O), 1614, 1572, 1520, 1448 (aromatic ring) cm^{-1}; ^{1}H NMR (pyridine-d_5, 400 MHz) δ: 3.71 (3H, s, OCH_3-3'), 3.83 (3H, s, OCH_3-6), 3.94 (1H, ddd, *J* = 12.7, 7.1, 3.5 Hz, H-9'b), 4.28 (1H, ddd, *J* = 12.7, 5.3, 2.3 Hz, H-9'a), 4.53 (1H, ddd, *J* = 8.0, 3.5, 2.3 Hz, H-7'), 5.55 (1H, d, *J* = 8.0 Hz, H-8'), 6.40 (1H, d, *J* = 9.6 Hz, H-3), 6.74 (1H, s, H-5), 7.25 (1H, dd, *J* = 7.1, 5.3 Hz, OH-9', D_2O exchangable), 7.30 (1H, d, *J* = 8.0 Hz, H-5'), 7.36 (1H, dd, *J* = 8.0, 2.0 Hz, H-6'), 7.43 (1H, d, *J* = 2.0 Hz, H-2'). 7.72 (1H, d, *J* = 9.6 Hz, H-4); ESI-MS *m/z* 409 $[M+Na]^+$

Cleomiscosin C (22): Whitish solid; IR υ_{max} (ATR) 3381 (OH), 1698 (C=O), 1612, 1573, 1460 (aromatic ring) cm^{-1}; ^{1}H NMR (pyridine-d_5, 400 MHz) δ: 3.79 (6H, s, OCH_3-3' and OCH_3-5'), 3.81 (3H, s, OCH_3-6), 3.94 (1H, br d, *J* = 13.8 Hz, H-9'b), 4.34 (1H, br d, *J* = 13.8 Hz, H-9'a), 4.52 (1H, dt, *J* = 8.1, 2.5 Hz, H-8'), 5.61 (1H, d, *J* = 8.1 Hz, H-7'), 6.46 (1H, d, *J* = 9.3 Hz, H-3), 6.75 (1H, s, H-

5), 7.22 (2H, s, H-2' and H-6'), 7.60 (1H, br s, OH-9', D_2O exchangable), 7.76 (1H, d, *J* = 9.3 Hz, H-4), 11.09 (1H, s, OH-4', D_2O exchangable); ESI-MS *m*/*z* 439 $[H+Na]^+$

Cleomiscosin D (23): Whitish solid; ^{1}H NMR (pyridine-*d*$_5$, 400 MHz) δ: 3.78 (6H, s, OCH_3-3' and OCH_3-5'), 3.83 (3H, s, OCH_3-6), 3.97 (1H, br d, *J* = 11.8 Hz, H-9'b), 4.30 (1H, br d, *J* = 11.8 Hz, H-9'a), 4.57 (1H, ddd, *J* = 8.0, 3.3, 2.1 Hz, H-7'), 5.56 (1H, d, *J* = 8.0 Hz, H-8'), 6.41 (1H, d, *J* = 9.4 Hz, H-3), 6.74 (1H, s, H-5), 7.21 (1H, s, H-2' and H-6'), 7.74 (1H, d, *J* = 9.4 Hz, H-4); ESI-MS *m*/*z* 439 $[M+Na]^+$

Malloapelin A (24): Yellowish solid; IR υ_{max} (ATR) 3354 (OH), 1698 (C=O), 1619, 1575, 1523 (aromatic ring) cm^{-1}; ^{1}H NMR (CD_3OD, 400 MHz) δ: 3.57 (1H, dd, *J* = 12.8, 4.0 Hz, H-9'b), 3.86 (1H, dd, *J* = 12.8, 2.5 Hz, H-9'a), 3.87 (3H, s, OCH_3-3'), 4.18 (1H, ddd, *J* = 8.0, 4.0, 2.5 Hz, H-8'), 5.00 (1H, d, *J* = 8.0 Hz, H-7'), 6.27 (1H, d, *J* = 9.8 Hz, H-3), 6.64 (1H, s, H-5), 6.64 (1H, d, *J* = 1.6, H-6'), 6.67 (1H, d, *J* = 2.0 Hz, H-2'), 7.80 (1H, d, *J* = 9.8 Hz, H-4); ESI-MS *m*/*z* 389 $[M+H]^+$

Malloapelin B (25): Whitish solid; IR υ_{max} (ATR) 3342 (OH), 1696 (C=O), 1618, 1575, 1520 (aromatic ring) cm^{-1}; ^{1}H NMR (CD_3OD, 400 MHz) δ: 3.62 (1H, dd, *J* = 12.4, 6.0 Hz, H-9'b), 3.70 (1H, dd, *J* = 12.4, 2.7 Hz, H-9'a), 3.86 (3H, s, OCH_3-3'), 4.22 (1H, ddd, *J* = 8.2, 6.0, 2.7 Hz, H-7'), 4.87 (1H, d, *J* = 8.2 Hz, H-8'), 6.26 (1H, d, *J* = 9.4 Hz, H-3), 6.60 (1H, d, *J* = 2.0, H-6'), 6.63 (1H, d, *J* = 2.0 Hz, H-2'), 6.65 (1H, s, H-5), 7.81 (1H, d, *J* = 9.4 Hz, H-4); ESI-MS *m*/*z* 389 $[M+H]^+$

***ent*-11-α-Hydroxy-3-oxo-13-*epi*-manoyl oxide (26)**: Whitish powder; $[\alpha]^{26}_D$: –41 (*c* 0.095, $CHCl_3$); IR υ_{max} (ATR) 3439 (OH), 1700 (C=O), 1124 (ether) cm^{-1}; ^{1}H NMR ($CDCl_3$, 400 MHz) δ: 1.01 (3H, s, H-20), 1.04 (3H, s, H-19), 1.11 (3H, s, H-18), 1.24 (3H, s, H-16), 1.27 (3H, s, H-17), 1.38 (1H, d, *J* = 10.0 Hz, H-9), 1.47 (2H, m, H-6b and H-7b), 1.58 (1H, m, H-12b), 1.62 (1H, m, H-5), 1.63 (1H, m, H-6a), 1.76 (1H, m, H-1b), 1.81 (1H, m, H-7a), 2.49 (2H, m, H-2), 2.50 (1H, m, H-12a), 2.52 (1H, m, H-1a), 4.17 (1H, ddd, *J* = 10.0, 9.6, 4.4 Hz, H-11), 4.96 (1H, br d, *J* = 11.2 Hz, H-15b), 5.08 (1H, d, *J* = 17.8 Hz, H-15a), 6.00 (1H, br dd, *J* = 17.8, 11.2 Hz, H-14); ^{13}C NMR ($CDCl_3$, 100 MHz) δ: 16.1 (C-20), 20.8 (C-6 and 19), 24.7 (C-17), 27.0 (C-18), 32.3 (C-16), 33.9 (C-2), 38.0 (C-10), 40.3 (C-1), 42.4 (C-7), 45.3 (C-12), 47.6 (C-4), 54.8 (C-5), 62.6 (C-9), 65.7 (C-11), 74.2 (C-13), 76.7 (C-8), 110.0 (C-15), 147.6 (C-14), 217.6 (C-3); ESI-MS *m*/*z*: 321 $[M+H]^+$

Excoecafolin D (27): Whitish solid; $[\alpha]^{26}_D$: –28 (*c* 0.39, pyridine); IR υ_{max} (ATR) 3364 (OH), 1692 (C=O) cm^{-1}; ^{1}H NMR (acetone-*d*$_6$, 400 MHz) δ: 0.94 (3H, d, *J* = 6.8 Hz, H-18), 1.68 (1H, dd, *J* = 3.6, 2.0 Hz, H-12b), 1.71 (3H, q, *J* = 1.2 Hz, H-19), 1.79 (3H, dd, *J* = 1.2, 0.6 Hz, H-17), 1.92 (1H, br t, *J* = 13.4 Hz, H-12a), 2.19 (1H, ddd, *J* = 13.4, 6.6, 3.6 Hz, H-11), 3.25 (1H, d, *J* = 2.0 Hz, H-8), 3.32 (1H, s, H-7), 3.44 (1H, dd, *J* = 7.2, 6.0 Hz, OH-20, D_2O exchangeable), 3.55 (1H, dd, *J* = 12.0, 7.2 Hz, H-20b), 3.82 (1H, s, OH-13, D_2O exchangeable), 3.93 (1H, dd, *J* = 12.0, 6.0 Hz, H-20a), 4.07 (1H, m, H-14), 4.09 (1H, m, H-10), 4.18 (1H, dd, *J* = 4.4, 0.8 Hz, H-5), 4.47 (1H, d, *J* = 4.4 Hz, OH-5, D_2O exchangeable), 4.71 (1H, d, *J* = 0.8 Hz, OH-9, D_2O exchangeable), 4.96 (1H, quintet, *J* = 1.2 Hz, H-16b), 5.06 (1H, br s, H-16a), 5.16 (1H, d, *J* = 5.2 Hz, OH-4, D_2O exchangeable), 5.30 (1H, s, OH-14, D_2O exchangeable), 7.62 (1H, quintet, *J* = 1.2 Hz, H-1); ESI-MS *m*/*z* 397 $[M+H]^+$

Agallochin I (28): Colorless oil; $[\alpha]^{26}_D$: –41 (*c* 0.215, MeOH); IR υ_{max} (ATR) 3382 (OH) cm^{-1}; ^{1}H NMR ($CDCl_3$, 400 MHz) δ: 1.00 (1H, m, H-5), 1.01 (3H, s, H-17), 1.03 (1H, m, H-11b), 1.08 (1H, m, H-14b), 1.13 (1H, m, H-9), 1.14 (3H, d, *J* = 7.0 Hz, H-18), 1.21 (1H, m, H-1b), 1.22 (2H, m, H-12), 1.37 (1H, t, *J* = 12.4 Hz, H-7b), 1.56 (1H, dd, *J* = 10.0, 2.4 Hz, H-14a), 1.63 (1H, m, H-11a), 1.72 (1H, m, H-2b), 1.87 (1H, dd, *J* = 12.4, 4.0 Hz, H-7a), 1.95 (1H, qd, *J* = 7.0, 2.4 Hz, H-4), 2.03 (1H, dt, *J* = 13.2, 3.3 Hz, H-2a), 2.06 (1H, dt, *J* = 12.0, 3.3 Hz, H-1a), 3.84 (2H, d, *J* = 3.2 Hz, H-19), 3.82 (1H, d, *J* = 1.2 Hz, H-6), 5.50 (1H, d, *J* = 5.6 Hz, H-16), 5.56 (1H, d, *J* = 5.6 Hz, H-15); ^{13}C NMR ($CDCl_3$, 100 MHz) δ: 19.3 (C-18), 20.8 (C-11), 24.5 (C-17), 27.5 (C-2), 31.6 (C-1), 32.1 (C-12), 36.5 (C-10), 42.2 (C-4), 43.7 (C-13), 44.6 (C-9), 45.2 (C-7), 49.5 (C-8), 57.1 (C-5), 60.5 (C-14), 68.7 (C-19), 70.5 (C-6), 97.8 (C-3), 133.2 (C-15), 138.1 (C-16); ESI-MS *m*/*z* 305 $[M+H]^+$

(+)-Catechin (29): Whitish powder; $[\alpha]^{23}_{D}$: +130 (*c* 0.48, MeOH); IR υ_{max} (ATR) 3317 (OH), 1521, 1462 (aromatic ring) cm^{-1}; ^{1}H NMR (CD_3OD, 400 MHz) δ: 2.51 (1H, dd, *J* = 16.0, 2.0 Hz, H-4a), 2.85 (1H, dd, *J* = 16.0, 5.6 Hz, H-4b), 3.98 (1H, ddd, *J* = 7.6, 5.6, 2.0 Hz, H-3), 4.57 (1H, d, *J* = 7.6 Hz, H-2), 5.86 (1H, d, *J* = 2.4 Hz, H-6), 5.94 (1H, d, *J* = 2.4 Hz, H-8), 6.72 (1H, dd, *J* = 8.2, 2.0 Hz, H-6'), 6.77 (1H, d, *J* = 8.2 Hz, H-5'), 6.83 (1H, d, *J* = 2.0 Hz, H-2'); ESI-MS *m/z* 291 $[M+H]^+$

Kaempferol-3-*O*-β-D-glucoside (30): Yellowish solids; ^{1}H NMR (CD_3OD, 600 MHz) δ: 3.20 (1H, m, H-3''), 3.30 (1H, m, H-4''), 3.41 (1H, m, H-5''), 3.44 (1H, m, H-2''), 3.53 (1H, dd, *J* = 12.0, 5.4 Hz, H-6''b), 3.68 (1H, dd, *J* = 12.0, 2.4 Hz, H-6''a), 5.20 (1H, d, *J* = 7.2 Hz, H-1''), 6.17 (1H, d, *J* = 1.8 Hz, H-6), 6.35 (1H, d, *J* = 1.8 Hz, H-8), 6.89 (2H, dd, *J* = 9.0, 2.4 Hz, H-3' and H-5'), 8.05 (2H, dd, *J* = 9.0, 2.4 Hz, H-2' and H-6'); ESI-MS *m/z* 449 $[M+H]^+$

6'-(Stigmast-5-en-7-one-3-*O*-β-glucopyransidyl)hexadecanoate (31): Whitish solid; $[\alpha]^{23}_{D}$: –81 (*c* 0.10, $CHCl_3$); IR υ_{max} (ATR) 3371 (OH), 1732 (C=O), 1670 (C=O) cm^{-1}; ^{1}H NMR ($CDCl_3$, 400 MHz) δ: 0.67 (3H, s, H-18), 0.81 (3H, d, *J* = 7.2 Hz, H-26), 0.83 (3H, d, *J* = 7.6 Hz, H-27), 0.84 (3H, t, *J* = 6.8 Hz, H-16''), 0.87 (3H, t, *J* = 6.8 Hz, H-29), 0.92 (3H, d, *J* = 6.8 Hz, H-21), 1.18 (3H, s, H-19), 1.58 (2H, m, H-3''), 2.23 (1H, t, *J* = 11.4 Hz, H-8), 2.33 (2H, t, *J* = 7.6 Hz, H-2''), 2.59 (1H, m, H-12), 3.36 (1H, t, *J* = 8.8 Hz, H-4'), 3.36 (1H, t, *J* = 8.8 Hz, H-2'), 3.47 (1H, m, H-5'), 3.56 (1H, t, *J* = 8.8 Hz, H-3'), 3.66 (1H, m, H-3), 3.71 (1H, OH, D_2O-exchangeable), 3.86 (1H, OH, D_2O-exchangeable), 4.28 (1H, br d, *J* = 10.6 Hz, H-6'b), 4.39 (1H, br d, *J* = 7.6 Hz, H-1'), 4.40 (1H, br d, *J* = 7.6 Hz, H-6'a), 5.70 (1H, d, *J* = 0.8 Hz, H-6); ^{13}C NMR ($CDCl_3$, 100 MHz) δ: 11.9 (C-18 and C-29), 14.1 (C-16''), 17.2 (C-19), 18.9 (C-21), 19.0 (C-27), 19.8 (C-26), 21.2 (C-15), 22.7 (C-11 and C-15''), 23.0 (C-28), 25.0 (C-3''), 26.1 (C-23), 26.3 (C-16), 28.5 (C-2), 29.1 (C-25), 29.2~29.7 (C-4''~C-13''), 31.9 (C-14''), 33.9 (C-22), 34.2 (C-2''), 36.1 (C-20), 36.3 (C-1), 38.4 (C-10), 38.7 (C-4 & C-12), 43.1 (C-13), 45.4 (C-8), 45.7 (C-24), 49.88 (C-14), 49.90 (C-29), 54.7 (C-17), 63.3 (C-6′), 70.1 (C-4'), 73.4 (C-2'), 73.9 (C-5'), 76.0 (C-3'), 78.3 (C-3), 101.5 (C-1'), 126.3 (C-6), 164.8 (C-5), 174.5 (C-1''), 202.3 (C-7); ESI-MS *m/z*: 829 $[M+H]^+$

(6'-*O*-Palmitoyl)sitosterol-3-*O*-β-D-glucoside (32): Whitish solid; $[\alpha]^{22}_{D}$: –50 (*c* 0.28, $CHCl_3$); IR υ_{max} (ATR) 3401 (OH), 1736 (C=O) cm^{-1}; ^{1}H NMR ($CDCl_3$, 400 MHz) δ: 0.68 (3H, s, H-18), 0.81 (3H, d, *J* = 7.2, H-27), 0.84 (3H, t, *J* = 7.2, H-26), 0.86 (3H, t, *J* = 7.4, H-29), 0.88 (3H, t, *J* = 6.8, H-17''), 0.92 (3H, d, *J* = 6.4, H-21), 1.00 (3H, s, H-19), 2.34 (2H, t, *J* = 7.6, H-2''), 2.63 (1H, OH, D_2O-exchangeable), 3.05 (1H, OH, D_2O-exchangeable), 3.20 (1H, OH, D_2O-exchangeable), 3.37 (1H, m, H-2'), 3.39 (1H, m, H-4'), 3.45 (1H, m, H-5'), 3.53 (1H, m, H-3), 3.59 (1H, m, H-3'), 4.28 (1H, dd, *J*= 12.0, 2.0, H-6'b), 4.38 (1H, d, *J* = 7.6, H-1'), 4.40 (1H, dd, *J* = 12.0, 4.8, H-6'a), 5.36 (1H, d, *J* = 5.2, H-6); ^{13}C NMR ($CDCl_3$, 100 MHz) δ: 11.8 (C-18), 12.0 (C-29), 14.1 (C-17''), 18.8 (C-21), 19.0 (C-27), 19.3 (C-19), 19.8 (C-26), 21.0 (C-11), 22.7 (C-16''), 23.0 (C-28), 24.3 (C-15), 24.9 (C-3''), 26.0 (C-23), 28.2 (C-16), 29.1 (C-2), 29.2 (C-25), 29.2~29.7 (C-4''~C-14''), 31.8 (C-8), 31.9 (C-7 & C-15''), 33.9 (C-22), 34.2 (C-2''), 36.1 (C-20), 36.7 (C-10), 37.2 (C-1), 38.9 (C-4), 39.7 (C-12), 42.3 (C-13), 45.8 (C-24), 50.1 (C-9), 56.0 (C-17), 56.7 (C-14), 63.2 (C-6'), 70.0 (C-4'), 73.5 (C-2'), 73.9 (C-5'), 75.9 (C-3'), 79.6 (C-3), 101.2 (C-1'), 122.2 (C-6), 140.2 (C-5), 174.8 (C-1''); ESI-MS *m/z*: 837 $[M+Na]^+$

A mixture of β-sitosterol (33) and stigmasterol (34): Colorless needles (CH_2Cl_2-MeOH); IR υ_{max} (ATR) 3427 (OH) cm^{-1}; ^{1}H NMR ($CDCl_3$, 200 MHz) δ: β-sitosterol: 0.67 (3H, s, H-18), 0.81 (3H, d, *J* = 6.8 Hz, H-26), 0.83 (3H, d, *J* = 6.8 Hz, H-27), 0.84 (3H, t, *J* = 7.8 Hz, H-29), 0.91 (3H, d, *J* = 6.4 Hz, H-21), 1.00 (3H, s, H-19), 3.50 (1H, m, H-3), 5.34 (1H, br d, *J* = 5.2 Hz, H-6); stigmasterol: 0.69 (3H, s, H-18), 0.81 (3H, d, *J* = 6.8 Hz, H-26), 0.83 (3H, d, *J* = 6.8 Hz, H-27), 0.84 (3H, t, *J* = 7.8 Hz, H-29), 0.91 (3H, d, *J* = 6.4 Hz, H-21), 1.00 (3H, s, H-19), 3.50 (1H, m, H-3), 5.00 (1H, dd, *J* = 15.2, 8.0 Hz, H-23), 5.15 (1H, dd, *J* = 15.2, 8.0 Hz, H-22), 5.34 (1H, br d, *J* = 5.2 Hz, H-6)

A mixture of 3-*O*-β-D-glucopyranosyl β-sitosterol (35) & 3-*O*-β-D-glucopyranosyl stigmasterol (36): Whitish solid; IR υ_{max} (ATR) 3397 (OH) cm^{-1}; ^{1}H NMR (pyridine-d_5, 400 MHz) δ: 3-*O*-β-D-glucopyranosyl β-sitosterol: 0.67 (3H, s, H-18), 0.88 (3H, d, *J* = 7.6 Hz, H-27), 0.91

(3H, t, J = 8.2 Hz, H-29), 0.93 (3H, d, J = 8.4 Hz, H-26), 0.95 (3H, s, H-19), 1.04 (3H, d, J = 6.4 Hz, H-21), 2.48 (1H, t, J = 12.4 Hz, H-4b), 2.74 (1H, dd, J = 12.4, 2.6 Hz, H-4a), 3.96 (1H, m, H-3), 3.98 (1H, m, 5'), 4.06 (1H, t, J = 7.8 Hz, H-2'), 4.29 (2H, t, J = 5.2 Hz, H-3' & 4'), 4.42 (1H, dd, J = 12.0, 5.2 Hz, H-6'b), 4.57 (1H, dd, J = 12.0, 2.4 Hz, H-6'a), 5.06 (1H, d, J = 7.8 Hz, H-1'), 5.36 (1H, d, J = 4.8 Hz, H-6); 3-*O*-β-D-glucopyranosyl stigmasterol: 0.67 (3H, s, H-18), 0.88 (3H, d, J = 7.6 Hz, H-27), 0.91 (3H, t, J = 8.2 Hz, H-29), 0.93 (3H, d, J = 8.4 Hz, H-26), 0.95 (3H, s, H-19), 1.04 (3H, d, J = 6.4 Hz, H-21), 2.48 (1H, t, J = 12.4 Hz, H-4b), 2.74 (1H, dd, J = 12.4, 2.6 Hz, H-4a), 3.96 (1H, m, H-3), 3.98 (1H, m, H-5'), 4.06 (1H, t, J = 7.8 Hz, H-2'), 4.29 (2H, t, J = 5.2 Hz, H-3' & H-4'), 4.42 (1H, dd, J = 12.0, 5.2 Hz, H-6'b), 4.57 (1H, dd, J = 12.0, 2.4 Hz, H-6'a), 5.05 (1H, dd, J = 15.2, 8.4 Hz, H-22), 5.06 (1H, d, J = 7.8 Hz, H-1'), 5.24 (1H, dd, J = 15.2, 8.4 Hz, H-23), 5.36 (1H, d, J = 4.8 Hz, H-6)

Isopropyl *O*-β-(6'-*O*-galloyl)glucopyranoside (37): Yellowish solid; $[\alpha]^{26}_{D}$: –36 (c 0.25, MeOH); UV (MeOH) λ_{max} (log ε) 215 (4.38), 275 (4.01) nm; IR υ_{max} (ATR) 3320 (OH), 1693 (ester), 1609, 1535, 1448 (aromatic ring) cm^{-1}; ^{1}H NMR (CD_3OD, 400 MHz) δ: 1.18 (3H, d, J = 6.0 Hz, H-1), 1.20 (3H, d, J = 6.0 Hz, H-2), 3.18 (1H, m, H-3'), 3.37 (1H, m, H-4'), 3.39 (1H, m, H-5'), 3.55 (1H, m, 2'), 4.37 (1H, d, J = 7.6 Hz, H-1'), 4.38 (1H, dd, J = 12.0, 5.8 Hz, H-6'b), 4.52 (1H, dd, J = 12.0, 2.4 Hz, H-6'a), 7.07 (2H, s, H-1'' and H-3''); ^{13}C NMR (CD_3OD, 100 MHz) δ: 22.2 (C-1), 23.8 (C-2), 64.8 (C-6'), 71.8 (C-4'), 73.3 (C3), 75.1 (C-3'), 75.4 (C-2'), 78.0 (C-5'), 103.0 (C-1'), 110.2 (C-1'' and C-3''), 121.2 (C-2''), 146.5 (C-4'', C-6''), 139.9 (C-5''), 168.4 (C-7'); ESI-MS m/z 375 $[M+H]^+$

4-Hydroxy-3-methoxyphenol 1-*O*-β-D-(2',6'-di-*O*-galloyl)glucoside (38): Yellowish solid; $[\alpha]^{26}_{D}$: –41 (c 0.12, MeOH); UV (MeOH) λ_{max} (log ε) 271 (4.67), 278 (4.32) nm; IR υ_{max} (ATR) 3369 (OH), 1697 (ester), 1613, 1513, 1449 (aromatic ring) cm^{-1}; ^{1}H NMR (CD_3OD, 600 MHz) δ: 3.56 (3H, s, OCH_3-3), 3.58 (1H, dd, J = 9.6, 9.0 Hz, H-4'), 3.75 (1H, t, J = 9.0 Hz, H-3'), 3.80 (1H, ddd, J = 9.6, 6.6, 2.4 Hz, H-5'), 4.49 (1H, dd, J = 12.0, 2.4 Hz, H-6'b), 4.64 (1H, dd, J = 12.0, 6.6 Hz, H-6'a), 4.95 (1H, d, J = 8.1 Hz, H-1'), 5.12 (1H, dd, J = 9.0, 8.1 Hz, H-2'), 6.44 (1H, d, J = 2.8 Hz, H-2), 6.45 (1H, d, J = 7.8, 2.8 Hz, H-6), 6.57 (1H, d, J = 7.8 Hz, H-5), 7.13 (4H, s, H-2'', H-6'', H-2''', and H-6'''), ^{13}C NMR (CD_3OD, 150 MHz) δ: 56.2 (OCH_3-3), 64.8 (C-6'), 71.9 (C-4'), 75.5 (C-2'), 75.9 (C-5), 76.0 (C-3'), 103.2 (C-1'), 104.3 (C-2'), 110.3 (C-2'', C-6''or C-2''', C-6'''), 110.4 (C-2''', C-6''' or C-2'', C-6''), 110.8 (C-6), 116.1 (C-5), 121.3 (C-1'' or C-1'''), 121.4 (C-1''' or C-1''), 140.1 (C-4'' and C-4'''), 143.6 (C-4), 146.6 (C-3'', C-5'' or C-3''', C-5'''), 146.7 (C-3''', C-5'' or C-3'', C-5''), 149.2 (C-3), 152.7 (C-1), 167.8 (C-7'' or C-7'''), 168.3 (C-7''' or C-7''); ESI-MS m/z 629 $[M+Na]^+$

3-Methoxy-4-hydroxyphenyl 1-*O*-β-D-(6'-*O*-galloyl)glucopyranoside (39): Whitish solid; $[\alpha]^{26}_{D}$: –39 (c 0.335, MeOH); UV (MeOH) λ_{max} (log ε) 205 (4.42), 275 (4.02) nm; IR υ_{max} (ATR) 3281 (OH), 1693 (C=O), 1610, 1508, 1448 (aromatic ring) cm^{-1}; ^{1}H NMR (CD_3OD, 400 MHz) δ: 3.43 (1H, m, H-2'), 3.44 (1H, m, H-4'), 3.47 (1H, m, H-3'), 3.70 (3H, s, OCH_3-3), 3.70 (1H, m, H-5'), 4.42 (1H, dd, J = 12.0, 6.8 Hz, H-6'b), 4.59 (1H, dd, J = 12.0, 2.0 Hz, H-6'a), 4.72 (1H, d, J = 7.2 Hz, H-1'), 6.56 (1H, dd, J = 8.5, 2.5 Hz, H-6), 6.62 (1H, d, J = 8.5 Hz, H-5), 6.70 (1H, d, J = 2.5 Hz, H-2), 7.09 (2H, s, H-2'' and H-6''); ^{13}C NMR (CD_3OD, 100 MHz) δ: 56.3 (OCH_3-3), 65.0 (C-6'), 71.8 (C-4'), 75.0 (C-2'). 75.7 (C-5'), 77.8 (C-3'), 103.9 (C-2), 104.0 (C-1'), 110.17 (C-6), 110.21 (C-2'', C-6''), 116.1 (C-5), 121.4 (C-1''), 140.0 (C-4''), 143.1 (C-4), 146.6 (C-3'' and C-5''), 149.2 (C-3), 152.7 (C-1), 168.3 (C-7''); ESI-MS m/z 455 $[M+H]^+$

1,2,3,4,6-Penta-*O*-galloyl-β-D-glucose (40): Yellowish solid; $[\alpha]^{26}_{D}$: + 34 (c 0.36, MeOH); UV (MeOH) λ_{max} (log ε) 215 (4.98), 280 (4.64) nm; IR υ_{max} (ATR) 3355 (OH), 1695 (C=O), 1610, 1535, 1448 (aromatic ring) cm^{-1}; ^{1}H NMR (CD_3OD, 200 MHz) δ: 4.40 (1H, t, J = 9.8 Hz, H-6b), 4.45 (1H, t, J = 9.8 Hz, H-6a), 4.50 (1H, t, J = 9.8 Hz, H-5), 5.59 (1H, m, H-2), 5.62 (1H, m, H-4), 5.91 (1H, t, J = 9.8 Hz, H-3), 6.23 (1H, d, J = 8.2 Hz, H-1), 6.90 (2H, s, galloyl group), 6.95 (2H, s, galloyl group), 6.98 (2H, s, galloyl group), 7.05 (2H, s, galloyl group), 7.11 (2H, s, galloyl group); ESI-MS m/z 963 $[M+Na]^+$

Corilagin (41): Yellowish solid; $[\alpha]^{24}_{D}$: + 173 (*c* 0.5, acetone); UV (MeOH) λ_{max} (log ε) 215 (4.61), 270 (4.28) nm; IR υ_{max} (ATR) 3349 (OH), 1712 (C=O), 1610, 1518, 1447 (aromatic ring) cm^{-1}; ^{1}H NMR (acetone-d_6, 400 MHz) δ: 4.08 (1H, br s, H-2), 4.11 (1H, dd, *J* = 11.0, 8.2 Hz, H-6b), 4.47 (1H, br s, H-4), 4.52 (1H, t, *J* = 9.6 Hz, H-5), 4.84 (1H, br s, H-3), 4.97 (1H, t, *J* = 11.0 Hz, H-6a), 6.38 (1H, d, *J* = 1.6 Hz, H-1), 6.70 (1H, s, H-3'' or H-3'''), 6.85 (1H, s, H-3''' or H-3''), 7.13 (2H, s, H-2' and H-6'); ^{13}C NMR (acetone-d_6, 100 MHz) δ: 62.9 (C-4), 65.0 (C-6), 69.6 (C-2), 71.2 (C-3), 76.3 (C-5), 94.8 (C-1), 108.3 (C-3'' or C-3'''), 110.6 (C-3''' or C-3''), 111.6 (C-2' & 6'), 116.5 (C-1'' or C-1'''), 117.2 (C-1''' or C-1''), 121.5 (C-1'), 126.3 (C-2'' or C-2'''), 126.4 (C-2''' or C-2''), 137.4 (C-6'''), 137.9 (C-6''), 139.9 (C-4'), 145.4 (C-4'' or C-4'''), 145.6 (C-3' or C-5'), 146.0 (C-4''' or C-4''), 146.5 (C-5' and C-3'), 165.8 (C-7'), 167.8 (C-7''), 169.2 (C-7'''); ESI-MS *m/z* 657 [M+Na]$^{+}$

1,4,6-Tri-*O*-galloyl-β-D-glucose (42): Yellowish solid; $[\alpha]^{26}_{D}$: –34 (*c* 0.365, acetone); IR υ_{max} (ATR) 3390 (OH), 1704 (C=O), 1612, 1533, 1450 (aromatic ring) cm^{-1}; ^{1}H NMR (acetone-d_6, 400 MHz) δ: 3.73 (1H, t, *J* = 9.9 Hz, H-2), 3.99 (1H, t, *J* = 9.9 Hz, H-3), 4.14 (1H, ddd, *J* = 9.9, 5.2, 1.8 Hz, H-5), 4.19 (1H, dd, *J* = 12.3, 5.2 Hz, H-6b), 4.45 (1H, dd, *J* = 12.3, 1.8 Hz, H-6a), 5.26 (1H, t, *J* = 9.9 Hz, H-4), 5.85 (1H, d, *J* = 9.9 Hz, H-1), 7.13 (2H, s, H-2''' and H-6'''), 7.16 (2H, s, H-2'' and H-6''), 7.19 (2H, s, H-2' and H-6'); ^{13}C NMR (acetone-d_6, 100 MHz) δ: 64.1 (C-6), 72.3 (C-4), 74.6 (C-5), 74.9 (C-2), 76.3 (C-3), 96.2 (C-1), 110.8 (C-2''' and C-6'''), 111.0 (C-2'' and C-6''), 111.1 (C-2' and C-6'), 121.6 (C-1'), 122.2 (C-1''), 122.3 (C-1'''), 139.6 (C-4'''), 139.7 (C-4''), 140.1 (C-4'), 146.65 (C-3''' and C-5'''), 146.72 (C-3'' and C-5''), 146.8 (C-3' and C-5'), 166.1 (C-7'), 166.7 (C-7''), 167.1 (C-7'''); ESI-MS *m/z* 659 [M+Na]$^{+}$

1,3,6-Tri-*O*-galloyl-β-D-glucose (43): Yellowish solid; IR υ_{max} (ATR) 3354 (OH), 1702 (C=O), 1613, 1539, 1451 (aromatic ring) cm^{-1}; ^{1}H NMR (CD_3OD, 400 MHz) δ: 3.77 (1H, t, *J* = 8.9, Hz, H-2), 3.80 (1H, t, *J* = 8.9 Hz, H-4), 3.88 (1H, ddd, *J* = 8.9, 4.8. 2.5 Hz, H-5), 4.43 (1H, dd, *J* = 12.0, 4.8 Hz, H-6b), 4.58 (1H, dd, *J* = 12.0, 2.5 Hz, H-6a), 5.58 (1H, t, *J* = 8.9 Hz, H-3), 5.82 (1H, d, *J* = 8.9 Hz, H-1), 7.10 (2H, s, H-2''' and H-6'''), 7.14 (2H, s, H-2'' and H-6''), 7.16 (2H, s, H-2 and H-6); ESI-MS *m/z* 659 [M+Na]$^{+}$

Gallic acid 4-*O*-β-D-(6'-*O*-galloyl)-glucose (44): Yellowish solid; $[\alpha]^{26}_{D}$: –6 (*c*, 0.145, acetone); IR υ_{max} (ATR) 3289 (OH), 1699 (C=O), 1612, 1525, 1452 (aromatic ring) cm^{-1}; ^{1}H NMR (CD_3OD, 600 MHz) δ: 3.48 (1H, dd, *J* = 9.6, 7.8 Hz, H-3), 3.52 (1H, t, *J* = 9.6 Hz, H-4), 3.54 (1H, t, *J* = 7.8 Hz, H-2), 3.68 (1H, ddd, *J* = 9.6, 5.4, 2.4 Hz, H-5), 4.43 (1H, dd, *J* = 8.0, 5.4 Hz, H-6b), 4.61 (1H, dd, *J* = 8.0, 2.4 Hz, H-6a), 4.70 (1H, d, *J* = 7.8 Hz, H-1), 7.05 (2H, s, H-3' and H-5'), 7.12 (2H, s, H-2'' and H-6''); ^{13}C NMR (CD_3OD, 150 MHz) δ: 64.4 (C-6), 71.2 (C-4), 75.0 (C-2), 76.6 (C-5), 77.5 (C-3), 107.5 (C-1) 110.2 (C-3' and C-5'), 110.3 (C-2'' or C-6'')110.4 (C-6'' or C-2''), 121.3 (C-4' and C-1''), 137.5 (C-1'), 140.0 (C-4''), 146.5 (C-3'' and C-5''), 151.1 (C-2' and C-6'), 168.4 (C-7''), 174.9 (C-7'); ESI-MS *m/z* 485 [M+H]$^{+}$
